# Effectiveness of structured exercise program on insulin resistance and quality of life in type 2 diabetes mellitus–A randomized controlled trial

**DOI:** 10.1371/journal.pone.0302831

**Published:** 2024-05-21

**Authors:** Sampath Kumar Amaravadi, G. Arun Maiya, Vaishali K., B. A. Shastry

**Affiliations:** 1 Department of Physiotherapy, School of Sport, Exercise and Rehabilitation, University of Birmingham, Birmingham, United Kingdom; 2 Department of Physiotherapy, Manipal College of Health Professions, Manipal Academy of Higher Education, Manipal, Karnataka, India; 3 Department of Medicine, Kasturba Medical College, Manipal Academy of Higher Education, Manipal, Karnataka, India; UNAM Facultad de Estudios Superiores Zaragoza: Universidad Nacional Autonoma de Mexico Facultad de Estudios Superiores Zaragoza, MEXICO

## Abstract

**Objective:**

Impaired glucose control & Insulin resistance are reported to be risk factors for the development of cardiovascular diseases. To find the effects of a structured exercise program on insulin resistance, glycaemic control, functional capacity, and quality of life in patients with Type 2 diabetes mellitus.

**Design:**

Randomized, controlled trial.

**Setting:**

Diabetic Foot Clinic, Department of Physiotherapy & Department of General Medicine, Kasturba Hospital in Manipal, Karnataka, India.

**Participants:**

160 participants aged between 30–65 years with Type 2 diabetes mellitus.

**Intervention:**

A set of structured exercise programs (aerobic, resistance, and combined) along with the standard hospital care was performed 3–5 times weekly for 12 weeks.

**Measurements: Primary outcome measures:**

Fasting Insulin Level, Homa-IR, Six-minute walk test (6MWT), and WHOQOL-BREF questionnaire at baseline and 12th week.

**Secondary outcome measures:**

Body composition analysis, Fasting Blood Sugar, Postprandial Blood Sugar, Glycated Haemoglobin (HbA1c), and GPAQ questionnaire at baseline and 12th week.

**Results:**

Significant differences have been observed in Homeostasis model assessment for insulin resistance (Homa-IR) (F (1, 144) = 89.29, p < 0.001); Fasting insulin (FI) (F (1, 144) = 129.10, p < 0.001); Fasting blood sugar (FBS) (F (1, 144) = 12.193, p< 0.001); Post prandial blood sugar (PPBS) (F (1, 144) = 53.015, p< 0.001); glycated haemoglobin (HbA1c) (F (1, 144) = 80.050, p < 0.001); WHOQOL-Physical health (F (1, 144) = 20.008, p< 0.001), Psychological (F (1, 144) = 77.984, p< 0.001), Social relationship (F (1, 144) = 44.866, p< 0.001); Environmental (F (1, 144) = 69.974, p< 0.001); Six minute walk test (6MWT) (*F* (1, 144) = 84.135, *p*< 0.001) in the study group when compared with the control group from baseline to 12th week.

**Conclusions:**

The study reveals that a 12-week structured exercise training program effectively reduces insulin resistance, improves quality of life, enhances functional capacity, and improves glycaemic control in type 2 diabetes mellitus.

## Introduction

The American Diabetes Association (ADA) defines Diabetes Mellitus (DM) as a group of metabolic diseases characterized by hyperglycaemia resulting from defects in insulin secretion, insulin action, or both [1]. The prevalence of type 2 diabetes mellitus (T2DM) is increasing rapidly worldwide and parallels the increase in obesity prevalence. In 2019 prevalence of T2DM was 9.3% and is expected to increase to 10.9% by the year 2045 [2]. In T2DM, elevated glucose levels in circulating blood are caused by impaired glucose tolerance, which leads to the development of insulin resistance (IR).

T2DM complications are among the leading causes of morbidity and mortality. Long-term complications can be delayed by taking medications as prescribed along with a healthy lifestyle (i.e., diet and physical activity) [3]. Insulin resistance (IR) impairs the ability of muscle cells to take up and store glucose and triglycerides, which results in elevated levels of glucose and triglycerides circulating in the blood. Impaired glucose control and IR are risk factors for the development of cardiovascular diseases [4].

IR is commonly present in older adults, but has become increasingly prevalent at all ages, including middle-aged individuals who are overweight and sedentary [5]. IR is typically defined as decreased sensitivity and responsiveness to insulin-mediated glucose disposal and the inhibition of hepatic glucose production [6] IR plays a significant pathophysiological role in T2DM. It is commonly associated with visceral adiposity, glucose intolerance, hypertension, dyslipidaemia, endothelial dysfunction, and elevated levels of inflammatory markers [7].

Evaluation of insulin resistance and β-cell function is essential for understanding disease status. The gold standard for evaluating insulin sensitivity is the glucose clamp test (GCT) [8]. There are several techniques available for assessing insulin resistance, including the hyperinsulinemia-euglycemic glucose clamp, oral glucose tolerance test (OGTT), fasting insulin levels, glucose/insulin ratio, insulinogenic index (IGI), homeostatic model assessment, quantitative insulin sensitivity check index (QUICKI), minimal model analysis of frequently sampled intravenous glucose tolerance test, glucose insulin (GI) product, and fasting insulin resistance index (FIRI) [9]. However, many of these methods’ present difficulties for practical implementation in a clinical setting.

Insulin resistance can be evaluated using the homeostatic model assessment of insulin resistance (HOMA-IR), which is calculated from the levels of fasting plasma glucose and immunoreactive insulin (IRI) [10]. The Homeostasis Model Assessment (HOMA) score, which is widely used in clinical research, provides an estimation of basal insulin resistance (HOMA-IR) and β-cell function (HOMA-%B). This assessment is based on a mathematical model that uses fasting glucose and insulin levels to predict the expected response of β cells and insulin resistance [11].

Recently, healthcare providers have gained greater understanding of the significance of assessing and monitoring health-related quality of life in individuals with T2DM. Several studies have highlighted that individuals with T2DM often experience diminished quality of life, suboptimal glycaemic control, and decreased functional capacity [12–14]. This growing awareness has led to an increased emphasis on evaluating and addressing these aspects of well-being in T2DM patient care.

The first approach to managing hyperglycaemia typically involves lifestyle interventions, including dietary adjustments and promoting weight loss through exercise, which are crucial for the management of type 2 diabetes mellitus (T2DM) [15]. Weight loss has been shown to have beneficial effects on metabolic control and cardiovascular risk factors in individuals with T2DM. Successful long-term weight control programs often incorporate a combination of diet, exercise, and lifestyle modification [16, 17].

Exercise and physical activity are considered cornerstones in the prevention and treatment of diabetes [18]. Exercise improves glycaemic control, even in the absence of weight loss, and results in reduced body fat content and an increased insulin response [19] Both aerobic and resistance exercises effectively improved insulin sensitivity and led to better glycaemic control in patients with type 2 diabetes. Aerobic exercise has been extensively investigated and has been shown to be beneficial for glucose lipid metabolism. Resistance training has known benefits for older patients with impaired glucose levels [20]. Earlier reviews have established that aerobic and resistance training can be used to improve the regulation of glucose as well as provide a synergistic effect when combined in a structured exercise program across all ages [21].

It has been reported that exercise intervention with a 24-week program has a beneficial effect on patients with T2DM, resulting in increased cardiovascular fitness and reduced BMI [18]. In an earlier study, the type and duration of exercise had a more significant effect on the results. However, in most studies, the effect of exercise on insulin resistance has not been sufficiently assessed. This study aimed to assess the effects of exercise on insulin resistance and quality of life in patients with T2DM.

## Materials and methods

### Design

We conducted a 12-week, single-centre, randomized, controlled trial from November 2016 to June 2019. The study was approved by the Institutional Ethics Committee (IEC:339/2016) to conduct the study from 14/06/2016 to 13/06/2019. Also, the committee performed detailed interim monitoring. It was mandatory to report to the IEC if any adverse effects were notified in participants. After obtaining the institutional ethical permission in June 2016, at once the study was submitted for trial registration which registered retrospectively with the Clinical Trial Registry of India (reference no: CTRI/2016/11/007488) on 24/11/2016. However, during this ongoing process we started screening the participants and officially enrolled them into the study after trial registered. All the participants provided informed consent. The authors confirm that all ongoing and related trials for this intervention are registered.

### Setting

Exercise intervention was performed at the Diabetic Foot Clinic, Department of Physiotherapy, Department of Medicine, Kasturba Hospital, Manipal, Karnataka, India.

### Participants

Eligible participants for this study included individuals who had been diagnosed with type 2 diabetes mellitus and were currently receiving treatment with oral hypoglycaemic agents with or without insulin therapy. The age range for inclusion was 30–65 years, and both males and females were eligible to take part. Participants meeting any of the following criteria were excluded from the study: type 1 diabetes mellitus, confirmed respiratory disease, coronary artery disease, neurological disorders, musculoskeletal problems that could hinder exercise training, uncontrolled hypertension (systolic blood pressure > 180 mmHg or diastolic blood pressure > 120 mmHg), pregnancy, thyroid disorder, or lack of willingness to participate in the study. The purpose of the study and the benefits of participation were explained to the prospective participants. The participants were asked to read an information sheet and sign a written informed consent form.

**Fig 1:**
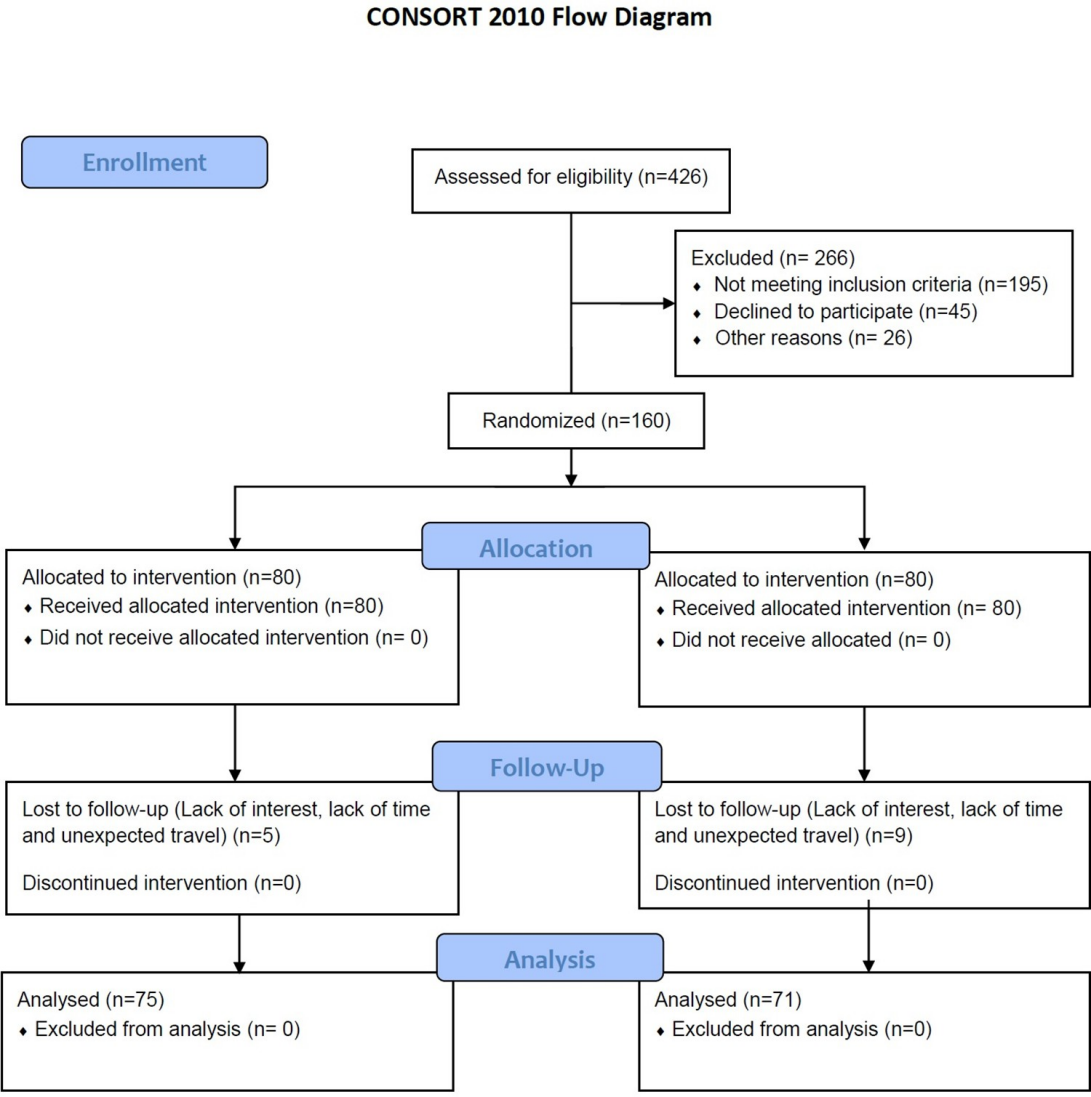
Consort flowchart of participants.

A total of 426 participants underwent screening, and 160 eligible participants were ultimately recruited based on meeting the inclusion and exclusion criteria. (Fig 1) Initial screening involved the use of the Physical Activity Readiness Questionnaire (PAR-Q) [22] and American Heart Association (AHA)/American College of Sports Medicine (ACSM) risk stratification questionnaire [23].

### Randomization

Purposive sampling is used in this study. Following evaluation by a physiotherapist (principal investigator), the participants were randomly assigned to either Group A or Group B. Group A, consisting of 80 participants, served as the study group, following an individually tailored structured and supervised exercise program. On the other hand, Group B, also including 80 participants, served as the control group. Block randomization was used to allocate participants to either group. The sequence was decided using randomly permuted blocks of equal size, with each block containing ten allotments. Sixteen blocks were arranged, with ten envelopes in each block. Randomization was performed using Sequentially Numbered Opaque Sealed Envelopes (SNOSE), where the principal investigator generated the sequence and assigned participants to groups based on the number retrieved from the envelope. Assessment and exercise prescriptions were provided free of charge to all study participants. The study involved 16 blocks, with 10 participants per block.

### Intervention

The study group underwent a 12-week intervention given by principal investigator consisting of an individualized and structured exercise program in addition to receiving standard hospital care. The exercise program included walking and active exercises for both the upper and lower limbs. The prescribed exercises were decided based on the evaluation of the 6-minute walk distance (6 MWD) and the rating of perceived exertion (RPE) achieved during the test. During the first visit, exercises were demonstrated to the patient and taught to both the patient and their caregiver. The patient education manual (S1 File) served as a reference.

During the first two weeks, all study participants underwent a supervised structured exercise program within the hospital setting. Following this period, they were prescribed a structured exercise-based rehabilitation program, which included progressing exercises at weeks 2nd and 6th week. A review and reassessment were conducted after 12 weeks. The exercise sessions began with a 10-minute warm-up session consisting of light intensity exercises such as breathing exercises, upper limb exercises, heel rise, and stretching of the tendo Achilles, hamstring, and quadriceps muscles. This was followed by a minimum of 20 min of walking on a treadmill at a moderate intensity and concluded with a 10-minute cool-down period. All the participants underwent moderate-intensity exercises.

All exercises were performed at a rate of 4–6 out of 10 on the rating of perceived exertion (RPE) scale. The patients were advised to exercise at least 3–5 times per week. The participants were taught to recognize the signs of hypoglycaemia and proper immediate measures. Exercise termination criteria were provided, recommending that participants stop exercising if they experienced dyspnoea, chest pain, breathlessness, or signs of hypoglycaemia. Aerobic exercise, specifically walking, was initially started at 15–20 minutes per session and later progressed to 30–45 minutes per session. At the six-week mark, the intensity or duration of exercise was adjusted based on the 6-minute walk distance (6 MWD) evaluation. A patient education manual was developed to provide detailed information on all the prescribed exercises. The book includes images that serve as a guide for patients to perform exercises at home (S1 File).

Walking intensity was decided by the individual’s intensity and heart rate response observed during the baseline 6-minute walk test (6 MWT). Patients were instructed to walk while maintaining a rating of perceived exertion (RPE) between 12–13 out of 20 or 4–6 out of 10. The duration of the walking sessions was progressively increased to achieve at least 30 minutes of walking time per day. In addition to the walking program, patients were taught exercises targeting the major muscle groups of the trunk, shoulder girdle, pelvic girdle, and muscles of the upper and lower limbs.

Initially, patients were prescribed a starting point of five repetitions with 1–2 sets of exercises, which gradually progressed every two weeks in terms of both sets and repetitions. At the conclusion of each exercise session, patients were instructed to perform a 5-10-minute cool-down period. The duration of each exercise session initially ranged from to 10–20 minutes, with a gradual progression to 30–60 minutes by the end of the 12-week period. The progression of exercises involved increasing the number of repetitions and extending the duration of walking while maintaining the target rating of perceived exertion (RPE) between 12–13 out of 20 or 4–6 out of 10.

All patients were monitored and followed up for 12 weeks, with a re-evaluation conducted at the end of this period. They received education about the termination criteria for exercise, which included symptoms such as increased shortness of breath, fainting, palpitations, and chest pain. Patients were recommended to seek immediate medical attention if any of these symptoms were present. To track exercise adherence, an exercise logbook was provided, and weekly phone calls were made to the patients during their participation in the structured exercise program. Adherence to the program was decided based on the completion of > 70% of the scheduled exercise sessions. The exercise protocol used in this study was developed based on an earlier study conducted by Maiya et al. [24] Additionally, the guidelines recommended by the American College of Sports Medicine (ACSM) were considered during the design of the protocol S2 File [25]. The participants in the control group did not receive a structured exercise program. Instead, they were recommended to continue with standard hospital care as per the recommendations. Both groups were assessed using outcome measures at baseline and 12 weeks. The quality-of-life questionnaire was administered by requesting patients to complete the questionnaire. For illiterate patients, the caregiver sought help.

### Outcomes and measurements

The primary outcome measures in this study included fasting insulin levels [mIU/L], Homeostatic Model Assessment of Insulin Resistance (HOMA-IR), and quality of life assessed using the World Health Organization Quality of Life-BREF (WHOQOL-BREF) questionnaire (Cronbach’s α = 0.73–0.81). The secondary outcome measures consisted of the six-minute walk test, body composition analysis, fasting blood sugar levels [mg/dL], postprandial blood sugar levels [mg/dL], HbA1c levels, and assessment of physical activity using the Global Physical Activity Questionnaire (GPAQ) (Cronbach’s α = 0.83–0.96). Both groups underwent assessment using these outcome measures at baseline and after the 12-week intervention period.

### Adverse events

In the present study participants were asked to document and record the adverse events in the exercise logbook. No adverse events were reported by participants in either group. The adherence of the participants to the exercise program was set at completion of >70% of the exercise protocol.

### Statistical analysis

The sample size was calculated from our pilot study using the mean change in fasting insulin levels [26].

N = 2[Z1-a2+Z1-β]2X σ2 (d)2

Z 1-α/2 = Level of significance Z 1-α/2 = 1.96

Z1-β = Power Z1-β = 0.84

σ = Standard deviation σ = 8.26

d = Clinical significance difference d = 4

Sample size n = 67

With a dropout rate of 20% and an added 13 participants in each group, a total sample size of n = 80 in each group was obtained. Data analysis was performed using SPSS version 20. Demographic details included the age, gender, medical history, known case of hypertension, were recorded and paired t test was used was used to calculate p value for baseline demographic data. Continuous variables were summarized using mean and standard deviations for age, duration of diabetes, blood pressure, body composition, vibration perception threshold (VPT), Homa-IR, FI (μu/mL), FBS (mg/dL), PPBS (mg/dL), HbA1c, WHOQOL and six-minute walk test. While categorical variables were presented as frequencies and percentages for gender, comorbidities,10-gram monofilament drug history and Global Physical Activity Questionnaire (GPAQ) score were presented in median (Interquartile range). In the present study the Kolmogorov–Smirnov test is used to determine the distribution of the data. For normally distributed data, repeated measures ANOVA was employed to compare the mean difference of an outcome between two groups over time, and the assumptions of the repeated measures ANOVA were checked using sphericity tests. In case of violation of assumption, the Greenhouse–Geisser correction was performed for outcomes wherever deemed necessary. Mann-Whitney U test was used to assess changes in GPAQ scores between the groups. The level of significance was set at p <0.05.

## Results

The CONSORT flow chart (Fig 1) explains the flow of participants enrolled in this study from baseline to the 12th week. We screened 426 subjects for eligibility, of which 266 were excluded because they did not fulfil the inclusion criteria. After exclusion, 160 participants were randomized into control and study groups. At the end of 12 weeks, there were 71 and 75 participants in the control and study groups, respectively. The various reasons for attrition were as follows: Attrition: Study group–05 [Lack of time–02; Travelling problem–03] Control group–09 [Lack of interest–04; Lack of time–02; Travelling problem -03].

The baseline demographic characteristics of the participants enrolled in this trial are shown in Table 1 and were homogenous at baseline. The body composition of the study participants was measured using bioelectrical impedance analysis (Omron Karada scan, model HBF-701), as illustrated in Table 2. Diabetic Neuropathy parameters of the foot, comorbidities, and drug history of the participants are presented in Tables 3–5, respectively.

**Table 1:** Demographic characteristics of the participants.

S.no	Variable	Study group (n = 75)	Control group (n = 71)	P value*
1	Age (years) **(Mean ± SD)**	56.05 ± 8.77	53.90 ± 10.20	0.17
2	Gender- male n (%) & female n (%)	58 (77.33) & 17(22.66)	39(54.92) & 32 (45.07)	-
3	Duration of diabetes (years) **(Mean ± SD)**	12.74 ± 8.67	11.46 ± 5.11	0.28
4	Systolic blood pressure (mmHg) **(Mean ± SD)**	140.84 ± 19.56	136.57 ± 11.50	0.11
5	Diastolic blood pressure (mmHg) **(Mean ± SD)**	81.97 ± 9.44	80.71 ±7.94	0.38

**Table 2:** Body composition of the participants.

S.no	Variable	Study group (n = 75)	Control group (n = 71)	P value*
		(Mean ± SD)	(Mean ± SD)	
1	Waist circumference in cm	92.12 ± 12.13	90.1 ± 9.4	0.27
2	Body mass index (BMI)in kg/m^2^	25.32 ± 3.16	26.27 ± 3.63	0.09
3	Total fat (%)	28.54 ± 5.12	28.26 ± 6.96	0.78
4	Visceral fat (%)	9.46 ± 4.26	10.12 ± 5.29	0.40
5	Free fat mass (%)	21.96 ± 4.03	23.26 ±4.80	0.07

*p<0.05, *95% Significance

**Table 3:** Diabetic neuropathy parameters of foot.

S.no	Variable	Study group (n = 75)	Control group (n = 71)
1	Vibration perception threshold (VPT) (volts)	Rt leg: 31.32 ± 12.61	Rt leg: 25.29 ± 11.27
	**(Mean ± SD)**	Lt leg: 33.46 ± 10.60	Lt leg: 27.11 ± 10.99
2	10-gram Monofilament	Present: 66 (88)	Present: 61 (86)
	Right leg **n (%)**	Absent: 09 (12)	Absent:10(14)
	Left leg **n (%)**	Present: 64 (85)	Present: 66(93)
		Absent:11 (15)	Absent:05 (7)

**Table 4:** Comorbidities of the participants.

S.no	Variable	Study group	Control group	Difference (%)	95% CI	Chi-squared	DF	P value*
		n = 75 (%)	n = 71 (%)					
1	Obesity	39(52)	42(59.2)	7.2	-8.75 to 22.61	0.76	1	0.38
2	Hypertension	53(70.6)	40(56.3)	14.3	-1.27 to 29.01	3.20	1	0.07
3	Ischemic heart disease	0	2(2.8)	2.8	-2.47 to 9.67	2.11	1	0.14
4	Eye complications [glaucoma, cataract, non-proliferative diabetic retinopathy (NPDR)]	8(10.7)	2(2.8)	7.9	-0.70 to 17.12	3.53	1	0.05

*p<0.05, *95% Significance

**Table 5:** Drug history of the participants.

S.no	Medications	Study group	Control group	Difference (%)	95% CI	Chi-squared	DF	P value*
		n = 75 (%)	n = 71 (%)					
1	OHA*	61 (81.3)	56(78.9)	2.4	-10.54 to 15.42	0.13	1	0.71
2	Insulin	1(1.3)	5(7.01)	5.71	-1.34 to 14.17	3.02	1	0.08
3	OHA + Insulin	12 (16)	6(8.5)	7.5	-3.50 to 18.40	1.88	1	0.17
4	Alternate medication	1(1.3)	4(5.6)	4.3	-2.44 to 12.33	2.04	1	0.15

*Oral hypoglycaemic agents–OHA; *p<0.05, *95% Significance

After 12 weeks of intervention, the study group shown significant improvements in various outcome measures, including HOMA-IR, fasting blood sugar, postprandial blood sugar, and glycated haemoglobin. The study participants in the study group received a tailored set of structured exercises, which consisted of aerobic exercises, resistance training with weights, and muscle stretching based on their first fitness assessment. Conversely, the control group received standard hospital care as recommended by the physician. The study group underwent follow-up at the 6th week, during which the exercises progressed. At the end of the 12-week period, all outcome measures were evaluated in both groups. Table 6 provides an overview of the impact of the structured exercise program on insulin resistance, as shown by HOMA-IR, fasting insulin, fasting blood sugar, postprandial blood sugar, and glycaemic control measured by glycated haemoglobin, comparing the baseline and 12th-week results for both the study and control groups.

**Table 6:** Effectiveness of structured exercise program on insulin resistance and glycaemic control between study and control group over 12 weeks.

S. No	Outcome measures	Sample (N) = 146	Baseline (Mean ± SD)	At 12 weeks (Mean ± SD)	Mean diff Post intervention units	Source	df	Error (Time)	Mean Square	F	P value*	Partial Eta Square
1	Homa IR	Study group	4.81 ± 2.69	3.35 ± 1.82	2.03	Time*Group	1	144	89.32	89.29	0.001	0.383
		(N = 75)										
		Control group	4.63 ± 2.69	5.38 ± 2.82								
		(N = 71)										
		Between the group	0.78	0.001								
		p value*										
2	FI (μu/mL)	Study group	12.67 ± 5.92	9.49 ± 4.64	3.56	Time*Group	1	144	315.476	129.107	0.001	0.473
		(N = 75)										
		Control group	12.15 ± 6.15	13.14 ± 6.39								
		(N = 71)										
		Between the group	0.62	0.001								
		p value*										
3	FBS (mg/dL)	Study group	159.55 ± 33.59	136.81 ± 18.11	18.2	Time*Group	1	144	5872.248	12.193	0.001	.078
		(N = 75)										
		Control group	159.69 ± 16.22	154.90 ±28.33								
		(N = 71)										
		Between the group	0.83	0.001								
		p value*										
4	PPBS (mg/dL)	Study group	210.2 ± 58.29	183.8 ± 44.1	17.7	Time*Group	1	144	28089.794	53.015	0.001	0.269
		(N = 75)										
		Control group	198.1 ± 48.3	201.5± 50.8								
		(N = 71)										
		Between the group	0.11	0.001								
		p value*										
5	HbA1c (%)	Study group	8.11± 1.27	7.52 ± 1.05	0.98	Time*Group	1	144	17.551	80.050	0.001	.357
		(N = 75)										
		Control group	8.06 ± 0.73	8.45 ± 0.80								
		(N = 71)										
		Between the group	0.43	0.001								
		p value*										

Homa-IR = Homeostasis model assessment for insulin resistance; FI- Fasting insulin; FBS Fasting blood sugar; PPBS- Postprandial blood sugar; HbA1c- Glycated haemoglobin.

*p<0.05, *95% Significance; 2-way mixed ANOVA- Time*group

Repeated measures ANOVA indicated significant timepoint*group interaction effect with Greenhouse–Geisser corrected for Homa-IR -F (1, 144) = 89.29, p < 0.001; FI- F (1, 144) = 129.10, p < 0.001, FBS- F (1, 144) = 12.193, p< 0.001; PPBS -F (1, 144) = 53.015, p< 0.001; and HbA1c - F (1, 144) = 80.050, p < 0.001 differed significantly between the study and control groups from baseline to 12th week.

Table 7 provides an overview of quality of life was assessed using the WHOQOL-BREF questionnaire, which includes of four domains. A repeated-measures ANOVA was conducted to examine the impact of the structured exercise program on post-intervention quality of life scores. Compared with the control group, the study group demonstrated significant improvements in the domains of physical health (F (1, 144) = 20.008, p < 0.001), psychological well-being (F (1, 144) = 77.984, p < 0.001), social relationships (F (1, 144) = 44.866, p < 0.001), and environmental factors (F (1, 144) = 69.974, p < 0.001) of the WHOQOL-BREF between baseline and the 12th week. These substantial changes show that exercise plays a crucial role in enhancing the quality of life of individuals with type 2 diabetes mellitus.

**Table 7:** Effectiveness of structured exercise program on quality of life between study and control group at baseline and the 12th week.

WHOQOL- BREF- Domains	Study group(n = 75)		% change	Control group(n = 71)		% change	P value*
	Pre (Mean ± SD)	Post (Mean ± SD)		Pre (Mean ± SD)	Post (Mean ± SD)		
Physical health	56.80 ± 11.23	65.16 ± 8.18	14.7	46.14 ± 6.73	47.81 ± 8.46	-3.6	<0.001
Psychological	51.40 ± 13.06	71.05 ± 11.27	38.2	43.16 ± 7.21	47.01 ± 7.74	8.9	<0.001
Social relationship	58.10 ± 18.91	77.36 ± 16.27	33.1	45.38 ± 12.11	45.05 ± 10.35	-0.7	<0.001
Environmental	59.81 ± 17.21	82.52 ± 13.05	37.9	42.67 ± 11.38	46.63 ± 9.45	9.2	<0.001

*p<0.05, *95% Significance

The functional capacity of the participants was assessed using the six-minute walk test, which is a standardized evaluation for measuring functional capacity in individuals with type 2 diabetes mellitus. Table 8 presents the six-minute walk distance values for both the control and study groups. Both groups proved homogeneity of functional capacity at baseline. After 12 weeks, the study group showed a significant increase of 146.59 meters, whereas the control group showed a slight increase of 45.63 meters. These findings showed that the 12-week structured exercise program had a significant impact (F (1, 144) = 84.135, p < 0.001). These results highlight the role of structured exercise programs in preserving and improving functional capacity.

**Table 8:** Pre-post six-minute walk distance (meters) at baseline and 12th week between study and control group.

Parameters	Study group(n = 75)			Control group(n = 71)		% change	P value*
	Pre (Mean ± SD)	Post (Mean ± SD)	% change	Pre (Mean ± SD)	Post (Mean ± SD)		
Six-minute walk distance (in meters)	534.37 ±122.94	680.96 ± 118.53	27.4	370.84 ± 76.93	416.47 ± 81.71	12.3	<0.001

*p<0.05, *95% Significance

Table 9 presents physical activity levels of the study participants were assessed using the Global Physical Activity Questionnaire (GPAQ). In addition to the structured exercise program, the study group participants received education about the importance of physical activity and reduction of sedentary behaviour, while the control group participants were instructed to continue with standard physician care. Both groups showed improvements in their physical activity levels (p < 0.001); however, the between-group analysis revealed a significant difference in physical activity level (Z = -2674, p = 0.008) between the control and study groups. At baseline, the study group consisted of 34 inactive (45.3%) and 41 active participants (54.7%). After 12 weeks of the structured exercise program, the number of inactive participants decreased to 20 (26.7%), while the number of active participants increased to 55 (73.3%) in the study group. This shows that, after the intervention, 14 (18.6%) participants in the study group became active. In contrast, the control group kept the same number of inactive participants (37 [52.1%]) and active participants (34 [47.9%]) from baseline to the 12th week due of the absence of exercise intervention.

**Table 9:** Physical activity level between study and control group at baseline and the 12th week.

Variable	Within group					Between group	
	Study group(n = 75)		Control group(n = 71)		P value*		
	Pre median (IQR)	Post Median (IQR)	Pre median (IQR)	Post Median (IQR)		Z	P value*
GPAQ in met.min. week	600(410, 720)	720 (540,1200)	510(360, 880)	540 (360,960)	<0.001	-2.674	<0.008

*p<0.05, *95% Significance

## Discussion

Our 12-week structured exercise program was effective in improving insulin resistance levels and quality of life in patients with T2DM. To the best of our knowledge, this is the first study to report the effects of a structured exercise program on IR and quality in patients with T2DM. The details of improvements in various outcome measures are discussed in the following sections.

### Effect of structured exercise program on insulin resistance in T2DM

In the present study, 12 weeks of structured exercise program was found to be effective in reducing insulin resistance and glycaemic levels in the study group as compared to the control group.

#### Homa-IR

In our study, implementation of a structured exercise program led to a notable mean reduction in HOMA-IR levels by 30.06% in the study group (p < 0.001). Conversely, the control group experienced an increase of 14.9% from baseline (Table 6). These findings are consistent with those of earlier studies that demonstrated similar outcomes. For instance, O’Donovan et al. [27] conducted a study investigating the effects of moderate and high-intensity exercise over 24 weeks, which resulted in a significant decrease of 66.6% in Homa-IR. Another study by Yorgi Mavros et al. [28] focused on insulin resistance and employed a 12-week intervention consisting of progressive resistance training three days a week, leading to a significant decrease in HOMA-IR levels.

In a study by Lazarevic et al. [29], the impact of aerobic exercise training on individuals with type 2 diabetes mellitus (T2DM) and insulin resistance was examined over a period of six months. The results revealed a decrease in Homa-IR levels of 19.1% at the third month and 41.22% at six months from baseline. Similarly, Kader et al. [30] conducted a study compared the effects of aerobic and resistance exercise training on insulin resistance in obese T2DM patients. The intervention lasted for three months, three days per week. Significant reductions in Homa-IR levels were seen, with decreases of 41.0% in the aerobic training group and 20.8% in the resistance training group. Ryoma Mischishita et al. [31] focused on overweight individuals with T2DM and found a decrease of 14.5% in Homa-IR levels. Additionally, an earlier study demonstrated that exercise therapy involving daily walking of 10,000 steps for six weeks resulted in a significant decrease of 29.1% in HOMA-IR levels. These findings highlight the consistent impact of exercise interventions on reducing insulin resistance in individuals with T2DM.

#### Fasting insulin

Fasting insulin (FI) is a crucial marker of insulin resistance. In our study, we saw a significant mean reduction of 24.34% in FI levels in the study groups (p<0.001). In contrast, the control group showed an increase of 8.11% from baseline (Table 6). These findings are consistent with those of earlier studies. Gary O’Donovan et al. [27] conducted a study that reported a notable decrease of 69.3% in the moderate-intensity exercise group and 73.7% after 24 weeks of exercise training. G. Lazarevic et al. [29] investigated the effects of aerobic exercise training alone and found a decrease of 1.36% at three months and 8.21% at six months when performed for 3–5 days a week. Similarly, Mischishita et al. [31] conducted a study involving overweight individuals with type 2 diabetes mellitus (T2DM) and reported a decrease of 11.36% in fasting insulin levels. These findings highlight the positive impact of exercise interventions on reducing fasting insulin levels and improving insulin resistance.

A study on the T2DM population by Akira A Katsuki et al. [32] found a 10.76% decrease in FI levels. In an earlier study, a decrease of 10.56% was reported after 16 weeks of aerobic exercise training alone [4]. On the other hand, a 12-week resistance exercise program on insulin resistance in T2DM by Geirsdottir et al. [20] found a significant decrease of 25.68% in fasting insulin level, which was consistent with our study findings.

### Effect of structured exercise program on glycaemic control in T2DM

The present study focused on the effect of a 12-week structured exercise program on glycaemic control with parameters such as FBS, PPBS, and HbA1c levels, which were found to be significantly reduced in the study group compared with the control group.

#### Fasting blood sugar (FBS)

In our study, we saw a significant mean reduction of 14.24% in fasting blood sugar (FBS) levels within the study group (p<0.001), whereas the control group displayed a slight increase of 0.28% from baseline (Table 6). These findings are consistent with those of the earlier studies. G. Lazarevic et al. [29] conducted a study on individuals with diabetes and found a reduction in FBS levels of 25.9% at three months and 39.8% at six months. Similarly, Mischishita et al. [31] investigated overweight individuals with type 2 diabetes mellitus (T2DM) and saw an 8.6% decrease in FBS levels after 12 weeks of exercise. Akira Akatsuki et al. [32] reported a significant decrease of 25% in FBS levels, whereas Anoop Misra et al. [33] found a reduction of 28.9% in FBS levels among Asian Indians with T2DM. Additionally, Bacchi et al. [34] conducted a study on the metabolic effects of aerobic and resistance training in T2DM subjects over four months and reported significant decreases of 15.2% and 12% in FBS levels in the respective exercise groups. These collective findings highlight the effectiveness of exercise interventions in reducing fasting blood sugar levels and improving glycaemic control in individuals with T2DM.

#### Postprandial blood sugar (PPBS)

In our study, we found that PPBS had a significant (p<0.001) mean reduction of 12.66% in the study group compared with the control group, where an increase of 6.60% from baseline was seen (Table 6). Our study findings are in line with the study done by Mischishita et al. [31] on overweight patients with T2DM, who found a decrease of 24.6% in PPBS after 12 weeks of exercise therapy.

#### Glycated haemoglobin (HbA1c)

HbA1c is the gold standard for the measurement of glycaemic control in diabetes, as it accurately reflects glycaemic control over time. It also predicts hyperglycaemia-related complications over time in patients with T2DM. Hence, any effort to lower HbA1c levels usually results in delayed microvascular-related complications in T2DM. In a clinical setting, type 2 diabetes usually presents with poor glycaemic control, which increases morbidity and mortality risk in the population [35].

The structured exercise program provided in the present study, along with standard care, had a glycation effect, which resulted in a mean reduction of HbA1c levels in the study group by 0.55 percent. We found a significant mean reduction of 6.80% in the study group, while HbA1c levels increased in the control group by 6.8% from baseline (Table 6). This possible change in HbA1c in the study group could be due to the combined effects of physician-prescribed management consisting of oral hypoglycaemic agents, insulin therapy, and a diabetic diet along with a structured exercise program comprising aerobic exercises, resistance exercise, flexibility, intrinsic foot muscle strengthening, and foot care.

Our findings are consistent with those in the existing literature. A Cochrane review by Thomas et al. [19] proved a reduction in HbA1c levels by 0.6% with exercise, which was both clinically and statistically significant. For studies with a duration of less than three months, the decrease was -0.8% and that of studies spanning less than six months, it was -0.7%, which is consistent with our study findings. Our findings are supported by Boule et al. [36], who reported that exercise reduces HbA1c levels by approximately 0.99%, which may be considered an adequate reduction to improve glycaemic control. In a study by Yavari et al. [37], a similar degree of reduction in HbA1c levels (0.73%) was found in the experimental group, while in the control group, the HbA1C values increased by 0.28%.

Several studies have compared the effects of aerobic and resistance exercises and concluded that aerobic exercises are more effective in reducing HbA1c levels. A systematic review by Umpierre et al. [38] reported a reduction in HbA1c levels of 0.73% and 0.57% with aerobic and resistance training, respectively. Similarly, another study found that HbA1c values decreased by 1.33%, 0.55%, and 1.74% in the aerobic, resistance, and combined exercise training groups, respectively, while it was 0.2% higher in the control group [39]. This improvement in HbA1c level may provide considerable benefit to patients with poor glycaemic control.

### Effect of structured exercise program on quality of life in T2DM

Based on our current study, the implementation of a structured exercise program for 12 weeks had a positive impact on the quality of life of patients with type 2 diabetes mellitus (T2DM). This is clear from the significant mean changes observed in the physical health (14.7%), psychological (38.22%), social relationship (33.14%), and environmental (37.9%) domains of the WHOQOL-BREF questionnaire (p < 0.001) when compared to the control group. Conversely, the control group shown mild changes of -3.6%, 8.9%, -0.7%, and 9.2% in the same domains (Table 7). Our study findings are consistent with the research conducted by Chew et al. [40] in T2DM patients, which further supports the positive influence of structured exercise programs on quality of life. Similarly, Bello et al. [41] assessed the effects of an 8-week aerobic exercise program on physiological parameters and quality of life in T2DM patients, reporting an overall significant change of 6.89%. These findings write down that participation in a 12-week structured exercise program can significantly enhance the quality of life of individuals with T2DM.

### Effect of structured exercise program on functional ability in T2DM

In our study, we measured the functional capacity of the participants using the six-minute walk test (6 MWT). An overall improvement of 146.59 meters (27.43%) was found in the study group at the end of the 12-week exercise intervention compared to baseline (Table 8). At present, the minimal clinically important difference (MCID) in the six-minute walk test is 50 meters,154 thus, this improvement in the study group was statistically significant (p<0.001). In contrast, in the control group, a minimal improvement of 46 months (46%) was seen, which was less than the MCID value for the 6 MWT.

Our findings align with those of earlier research conducted in this field. Lambers et al. [14] conducted a study investigated the effect of combined exercise training on obesity, diabetes, and cardiovascular risk in patients with type 2 diabetes mellitus. They reported a significant difference between the combination and endurance groups after three months of exercise training. Similarly, Tan et al. [42] conducted a study examined the effects of six months of combined aerobic and resistance training in patients with T2DM. They saw a significant improvement in walking distance of 58 m (39%), which is consistent with our findings. Patients with T2DM commonly show lower muscle strength [43, 44] and reduced skeletal mass in their lower limbs than individuals without diabetes [45]. This deficiency in muscle function may contribute to insulin resistance in these patients [46]. The 12-week structured exercise training program used in our study may help enhance muscle strength in elderly patients, thereby potentially addressing the issue of insulin resistance. 

### Effect of structured exercise program on physical activity in T2DM

Regular physical activity (PA) can improve blood glucose control and potentially delay T2DM onset. Studies show higher PA rates, moderate-intensity walking, and vigorous activity decrease T2DM risk, with higher fitness levels also associated with reduced risk. Sedentary behaviour and low PA are linked to non-communicable cardiovascular disorders, while consistent PA may delay T2DM development and reduce cardiovascular mortality risk in T2DM participants [47].

In the present study, we measured the PA of participants using the Global Physical Activity Questionnaire (GPAQ). We found a statistically significant difference of 20% in the study group and 5.8%, in the control group, which was significant within the groups. We also found a significant difference between the groups at baseline and 12 weeks (Table 9). A study conducted by Beverley et al. [48] proved an association between total activity and insulin sensitivity in both men and women. Furthermore, the study found that sedentary time, light activity, and activity intensity were also linked to insulin sensitivity. Several mechanisms have been proposed to explain the effect of physical activity (PA) on insulin sensitivity.

One theory suggests that PA enhances glucose transport into muscle cells by potentially increasing the expression of glucose transporter 4 [49]. Additionally, regular PA promotes a decrease in fat mass and an increase in lean mass, which expands the muscle available for glucose absorption [50]. Obesity stimulates the production of inflammatory cytokines, leading to insulin resistance [51]. In this context, reducing body weight through PA may serve as a mechanism for enhancing insulin sensitivity. Another mechanism is improved blood perfusion of muscles during PA, which can contribute to enhanced insulin sensitivity [52].

This study had several strengths. First, this is the first randomized controlled trial with a substantial sample size to evaluate the efficacy of a structured exercise program in improving insulin resistance and quality of life in individuals with type 2 diabetes mellitus (T2DM). Additionally, the exercise program implemented in this study was proven safe and possible for both clinical and research purposes in the target population. Furthermore, an important strength of the study is that the main outcome assessor responsible for assessing the Homa-IR was blinded, which helped minimize potential bias and enhance the validity of the findings.

Our study has some limitations. First, we did not record the post-exercise weight and BMI of the participants, which could have offered valuable insights into the potential impact of the exercise program on these measures. Second, comorbidities and drug effect can be indirect influencer which is partial limitation of the present study. Another limitation is that the individual dietary intake was not recorded, which could have influenced the outcomes of the study. It is important to acknowledge these limitations, as they may have an impact on the interpretation and generalizability of our findings.

## Conclusions

The present study suggests that a structured exercise rehabilitation program has a positive impact on the overall well-being and quality of life of individuals with type 2 diabetes mellitus. Consequently, this study concluded that a 12-week structured exercise training program effectively improves insulin resistance, quality of life, functional capacity, and glycaemic control in individuals with type 2 diabetes mellitus. These findings highlight the potential benefits of implementing such programs as part of the management and treatment of type 2 diabetes mellitus.

## Supporting information

S1 File Patient education manual. (PDF)

S2 File Exercise protocol. (PDF)

S1 Data (DOCX)
