# Supplementary material for: Effectiveness of structured exercise program on insulin resistance and quality of life in type 2 diabetes mellitus–A randomized controlled trial
Source: PLoS One. 2024 May 21;19(5):e0302831. doi: 10.1371/journal.pone.0302831 (PMC11108169; doi:10.1371/journal.pone.0302831)
Supplement: S1 File — (PDF) [file pone.0302831.s001.pdf]

**Supporting information:**

**S1: Patient education manual**

**MANIPAL**
ACADEMY *of* HIGHER EDUCATION
*(Deemed to be University under Section 3 of the UGC Act, 1956)*

**Title: Exercise in Type 2 Diabetes Mellitus**

**Principal Investigator: A Sampath Kumar,**
**Ph.D. scholar,**
**Department of Physiotherapy,**
**Manipal College of Health Professions,**
**Manipal Academy of Higher Education.**

---

**What is Type 2 Diabetes Mellitus?**

- Type 2 diabetes mellitus is characterized by elevated glucose levels in circulating blood, caused by impairment in glucose tolerance following the development of insulin resistance and relative insulin deficiency.

**Causes**

- It is caused by a combination of lifestyle and genetic factors

**Signs & Symptoms of diabetes mellitus-**

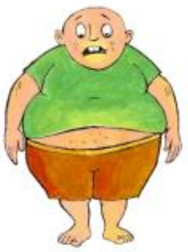

**Weakness & Tiredness**

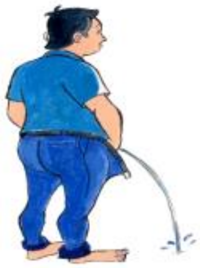

**Frequent urination**

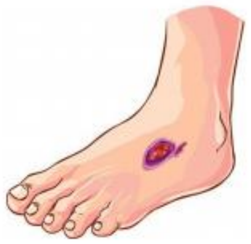

**Wounds that won't heal**

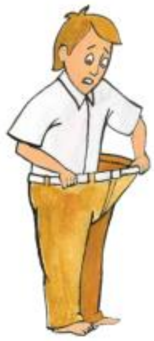

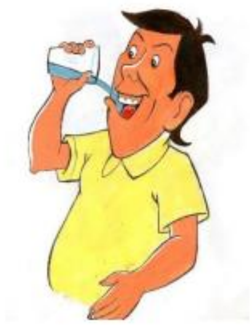

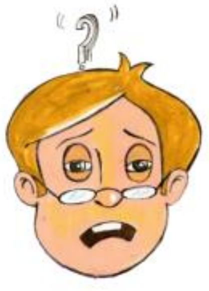

**Weight loss** **Thirsty** **Blurred vision**

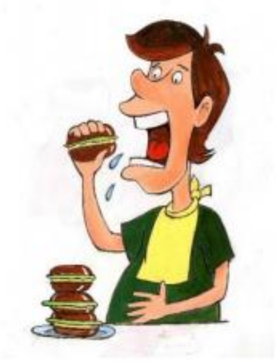

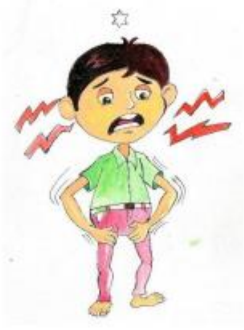

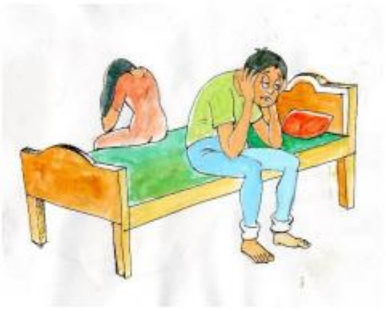

**Hungry** **Itching in genital** **Sexual problem**

**Complications with T2DM**

- Diabetes complications are the main causes of morbidity and mortality**.** It can be prevented by taking medication accurately with healthy diet and physical activity thus the long term complications will be delayed. They are categorised into acute and chronic complications

**Acute complications:** Diabetic Ketoacidosis , Hypoglycemia, Hyperglycemic Hyperosmolar State

**Chronic complications:**

| Microvascular | Macrovascular |
|---|---|
| Retinopathy | Coronary artery disease |
| Neuropathy | Peripheral arterial disease |
| Nephropathy | Cerebrovascular disease |

**Other complication:** Insulin Resistance, Diabetic Foot

**Insulin resistance (IR)**

- Insulin resistance occurs when insulin is produced by the body but not used effectively by the cells.
- IR impair the ability of muscle cells to take up and store glucose and triglycerides, which results in higher levels of glucose and triglycerides circulating in the blood.
- Impaired glucose control and insulin resistance are reported to be a risk factor for development of cardiovascular disease.

**Risk for Insulin Resistance-**

If you

- are overweight
- are physically inactive
- have a parent, brother, or sister with type 2 diabetes
- have polycystic ovary syndrome, also called PCOS
- have had gestational diabetes, which is diabetes that develops during pregnancy
- are age 45 or older
- have had above-normal blood glucose levels
- have high blood pressure

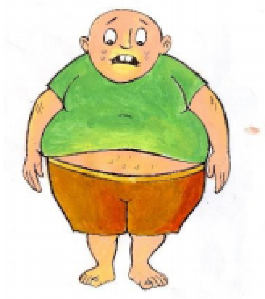

- have low HDL (good) cholesterol
- have high levels of blood fats called triglycerides
- Have had heart disease, a stroke, or disease of the blood vessels in your neck or legs.

**How is Insulin Resistance diagnosed**

- Various methods to measure Insulin Resistance are Hyperinsulinemic euglycemic glucose clamp, oral glucose tolerance test (OGTT), Fasting Insulin, Glucose/Insulin ratio, Insulinogenic index (IGI), Homeostatic model assessment (HOMA-IR).
- Most commonly used measures are Fasting Insulin and Homeostatic Model Assessment –Insulin Resistance (HOMA-IR).

## MANAGEMENT

**Triad of management:**

- Oral hypoglycemic agents (OHA)
- Insulin therapy
- Life style modifications
  1. Diet
  2. Exercise /Physical Activity

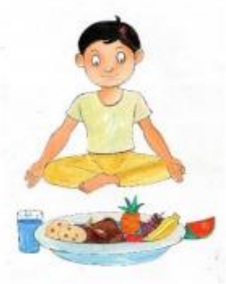

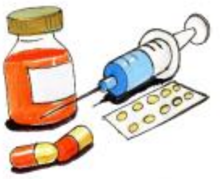

## Exercise & Type 2 Diabetes

**Benefits of exercise:**

- Helps prevent or delay the onset of type 2 diabetes.
- Exercise helps with:
  - Weight loss and maintenance
  - Stronger, healthier heart
  - Improved sleep
  - Improved mood, decreased stress, increased energy
  - Improved blood pressure, cholesterol, triglycerides, and blood glucose levels
  - Increased lean muscle mass and strength & injury prevention

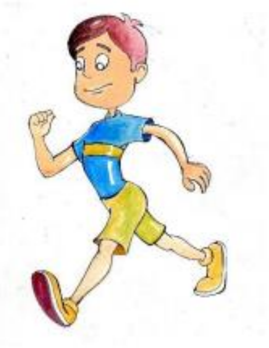

**Exercise & Blood glucose**:

- Exercise helps lower blood glucose by increasing the muscle's ability to take up and use glucose.
- Exercise can help lower the amount of medication needed to keep blood glucose levels within your goal range.
- Monitor blood glucose before and after you exercise to learn how your glucose level changes with activity.
- Stop exercising and test your blood glucose if you feel symptoms of hypoglycemia, including excessive sweating, anxiousness, shakiness, confusion, and/or low energy.
- A change of heart rate is normal when exercising.

## EXERCISE TRAINING

| Variable | Exercise prescription |
|---|---|
| Frequency | 3 days / week aerobic exercise<br>2 days / week of resistance exercise |
| Intensity | 40-85% HRR [Aerobic Exercise] , 60-80% 1 RM [Resistance Exercise]<br>RPE = 11-16 |
| Time | Aerobic: 30-60 mins per session<br>Resistance :8-12 reps/ exercise, 1-3 sets/exercise |
| Type of exercise | Aerobic : Brisk Walking , running , cycling<br>Resistance : Muscle groups- Plantar & Dorsiflexor, Quadriceps, Hip abductors , Hamstring, Biceps, Triceps & Deltoid<br>Flexibility: stretching exercises – Intrinsic foot muscle, Tendon Achilles, Hamstrings, Calf , wrist flexors and extensors |

# AEROBIC EXERCISES

## Treadmill walking

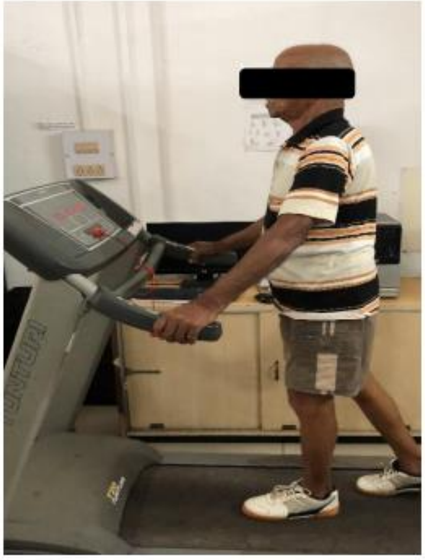

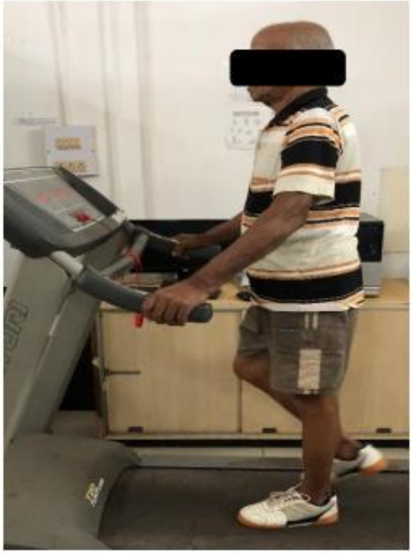

- **Brisk Walking:** The proper form for brisk walking is to stand up straight, bend your elbows and relax your shoulders. Look straight ahead and start walking, heal to toe. Move your arms forward and back as you walk.
- Brisk walking for 30 minutes at a moderate speed can help in burn 150 to 200 calories.

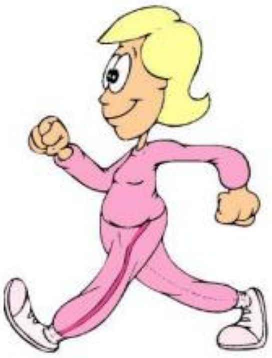

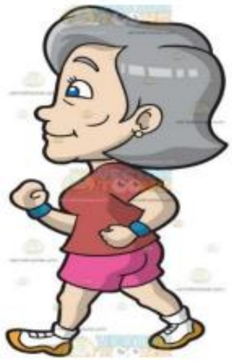

# RESISTANCE EXERCISES

## Thera band exercises

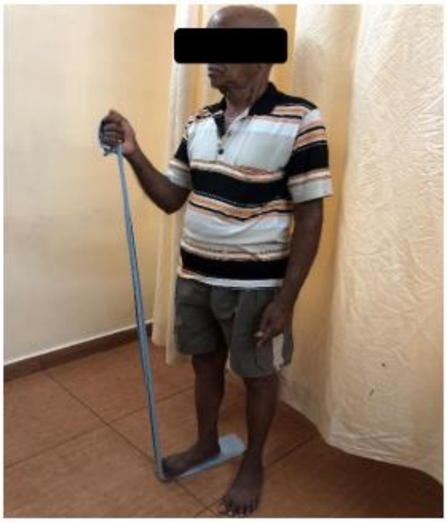
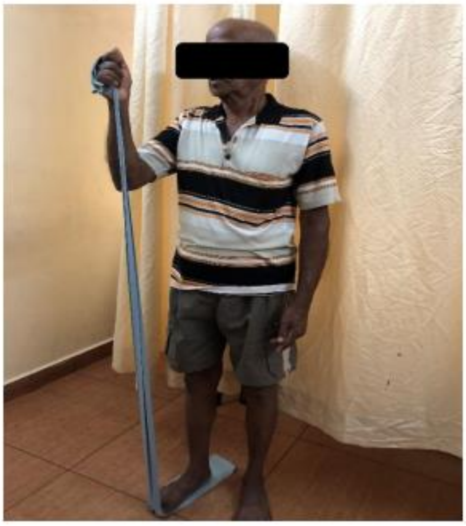

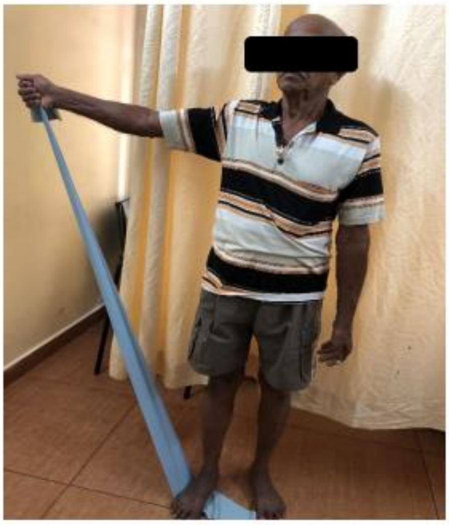
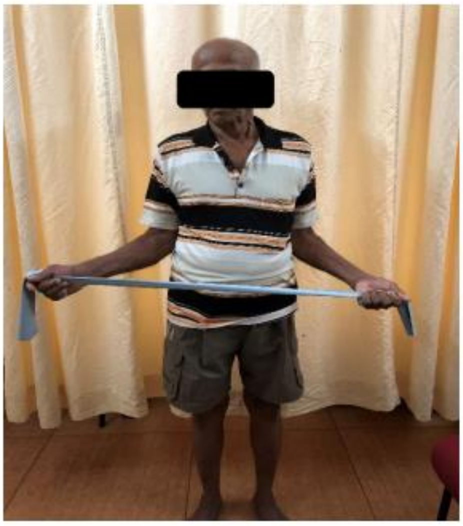

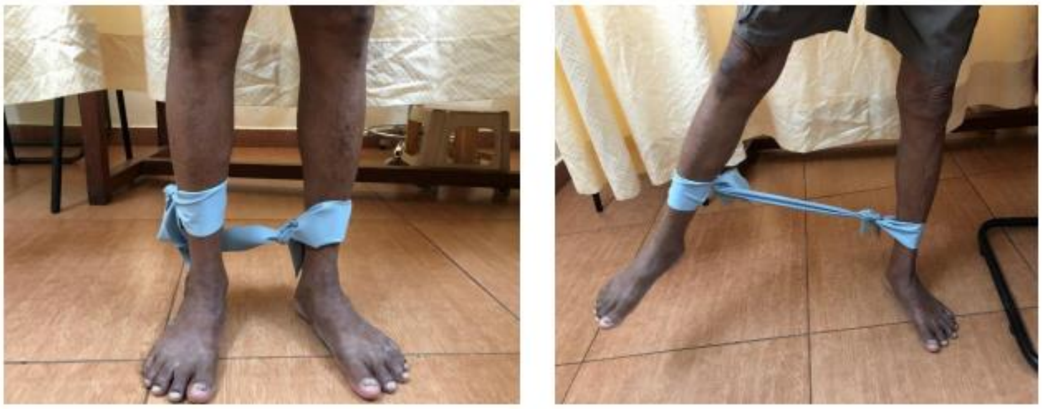

<u>**DYNAMIC QUADRICEPS**</u>

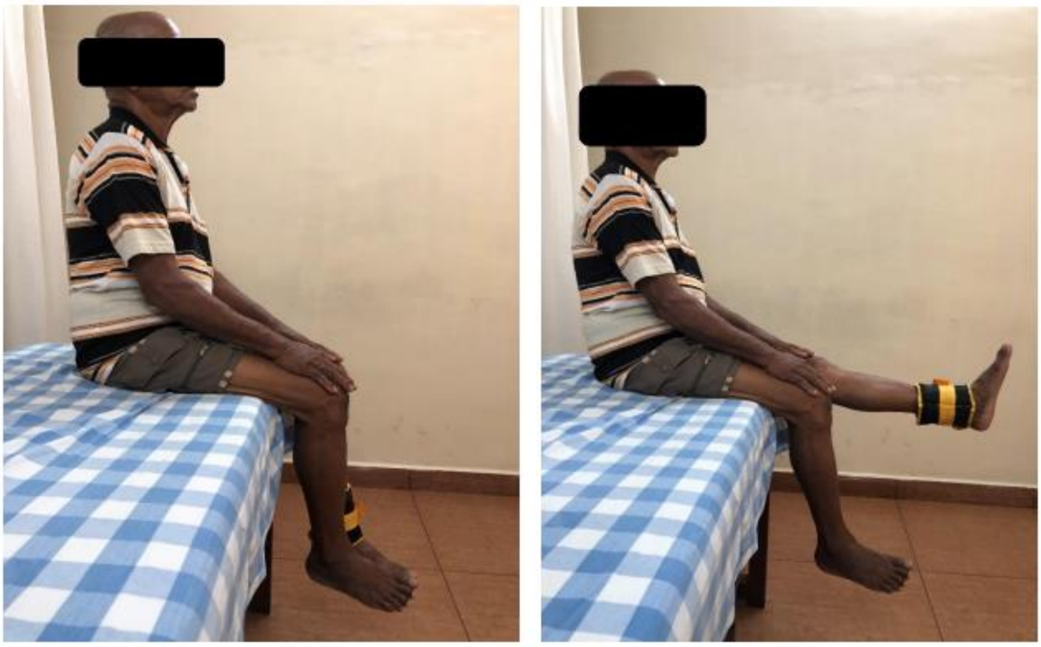

# KNEE EXERCISES

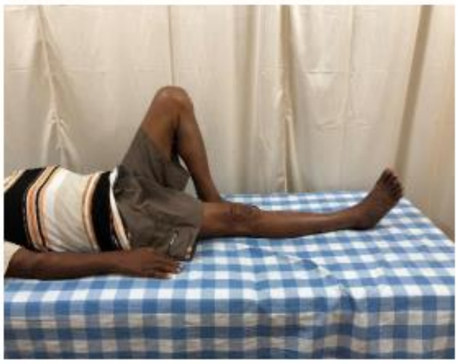

Knee -1

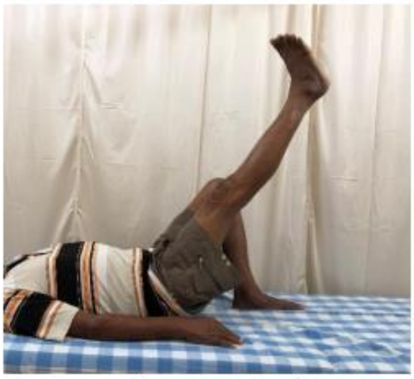

Knee-2

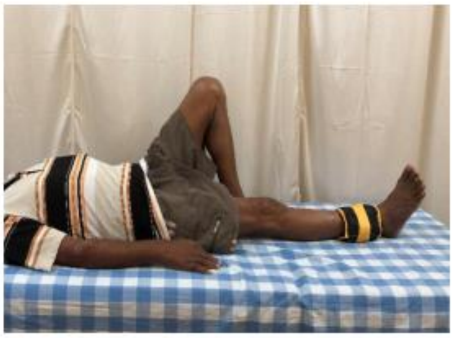

Knee -3 with 1 kg sand bag

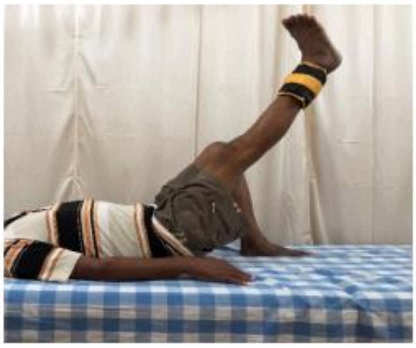

Knee -3 with 1 kg sand bag

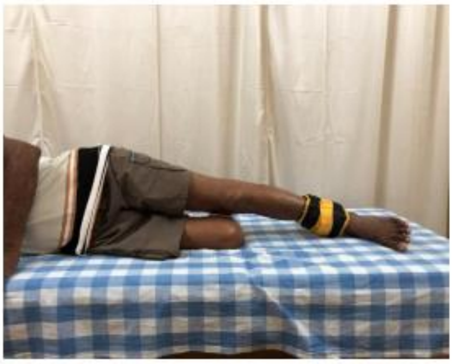

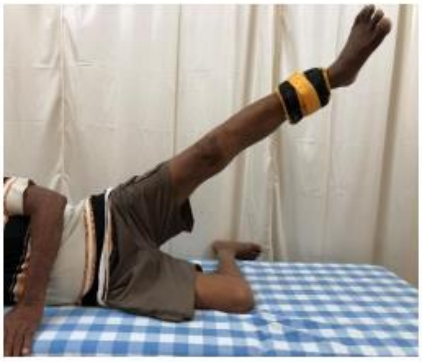

Side leg raise –with 1kg sand bag

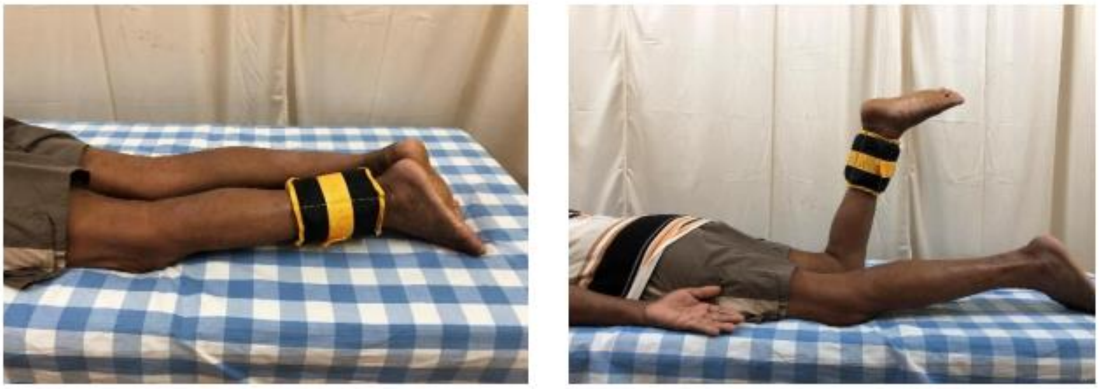

Knee bent with weight bag

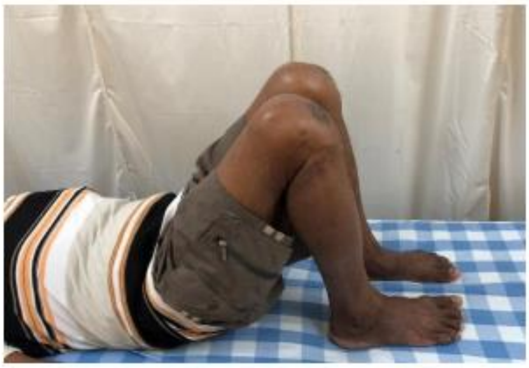

Toe - neutral position

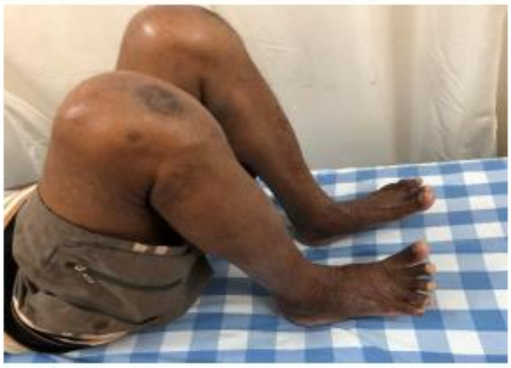

Toe raise

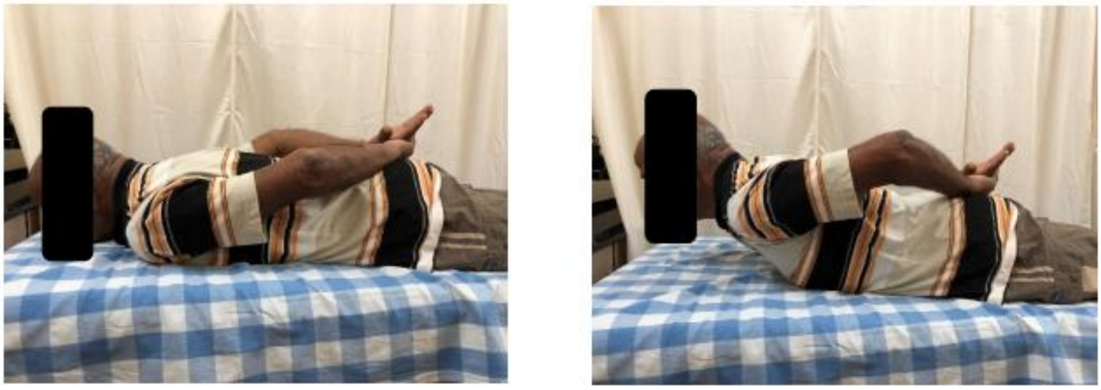

Chest raise

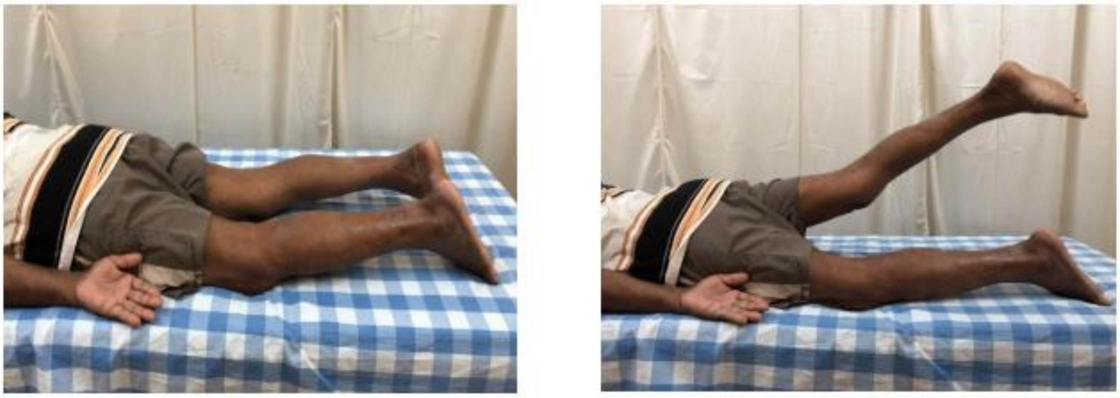

Straight leg raise

## FLEXIBILITY EXERCISES

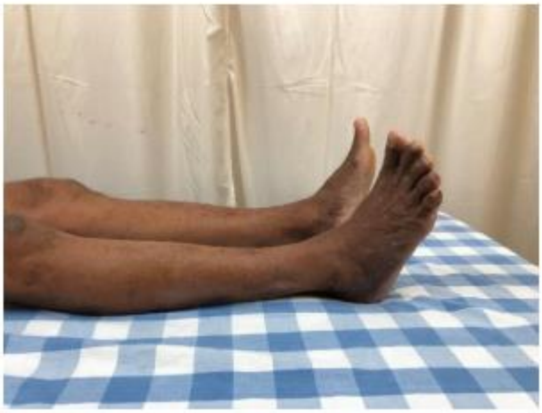

Neutral position

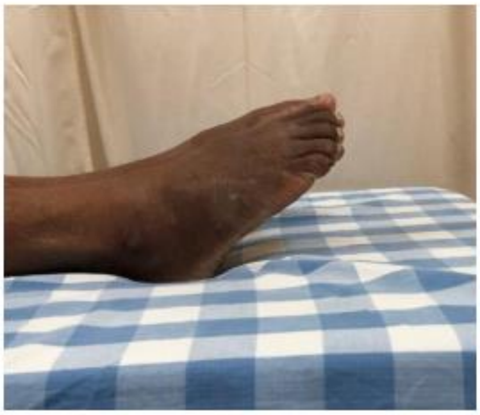

Dorsi flexion

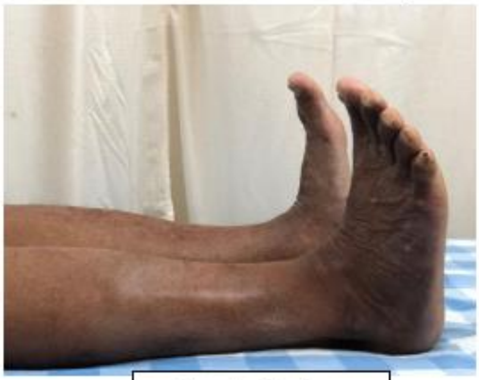

Plantar flexion

**BALL PRESS**

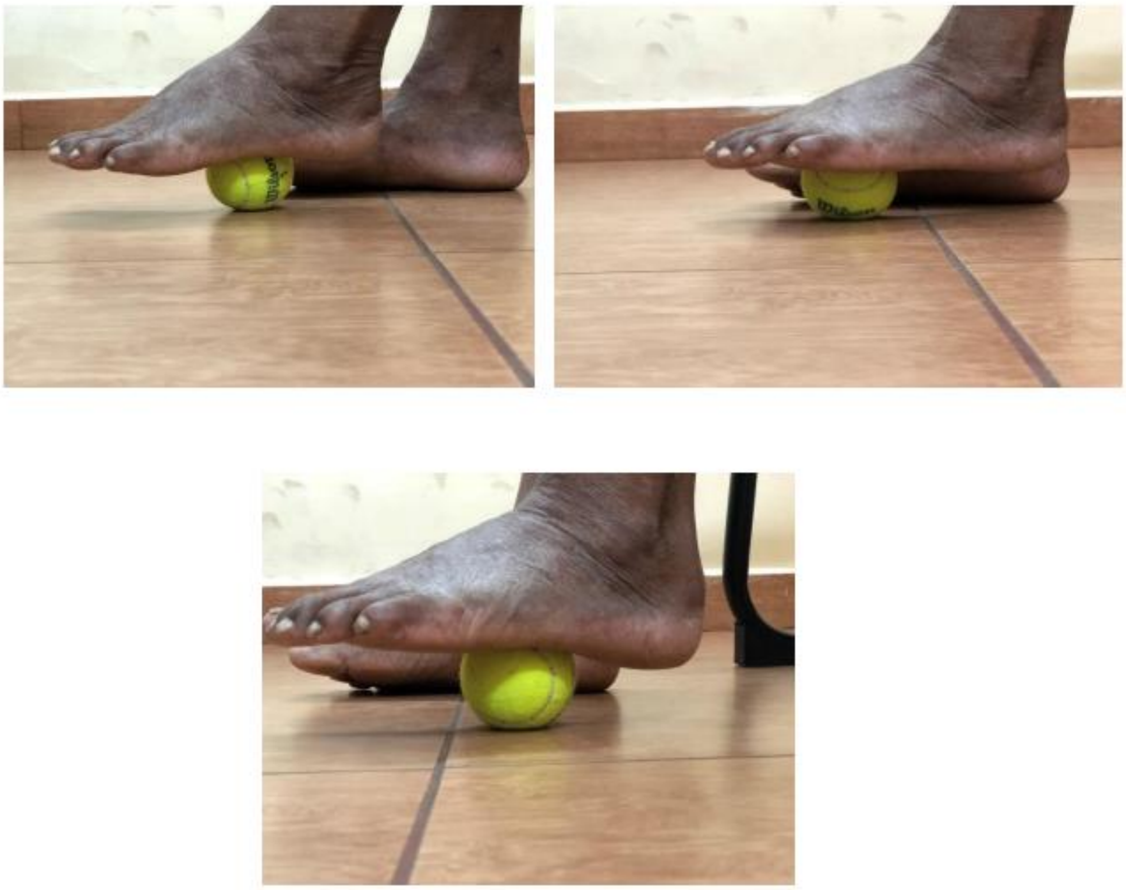

**INTRINSIC FOOT MUSCLE EXERCISES**

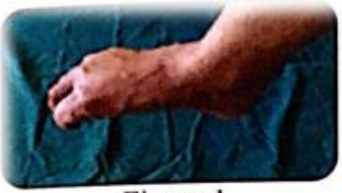
Figure 1

Step 1: Bend ankle and toes down.
(Fig. 1)

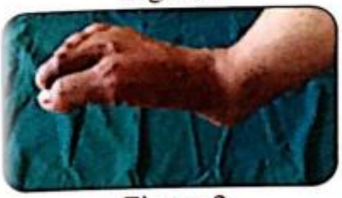
Figure 2

Step 2: Raise ankle up, but keep toes bent down (Fig. 2)

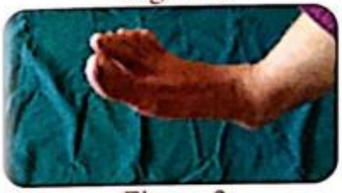
Figure 3

Step 3: Relax the toes (Fig. 3)

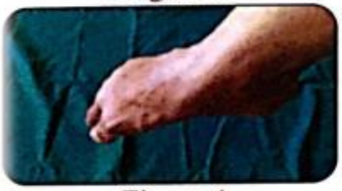
Figure 4

Step 4: Flex toes down and at the sam[illegible] time flex ankle down.
(Fig. 4)

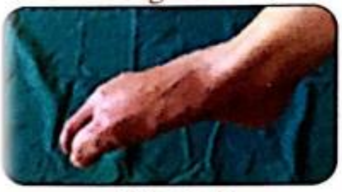
Figure 5

Step 5: While keeping toes flexed, move the foot inward (inversion) and then outward (eversion)
(Fig. 5 & 6)

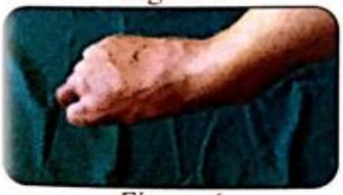
Figure 6

Repeat each exercise for
5- 10 repetitions per session

**Warning signs:**

- Chest discomfort
- Breathing difficulty
- Lightheadedness or Dizziness
- Discomfort in other areas of body
- Unusual sweating

**You should not exercise if:**

- Your blood sugar level is over 300 mg/dl
- You are sick
- You are short of breath
- You have ketones in your urine
- You are experiencing any tingling, pain or numbness in your legs


**Acknowledgement:**

- World Diabetes Foundation (WDF 15-941)- " Diabetic Foot Care: Stepping Ahead"
- Centre for Diabetic Foot Care & Research
- Mr. Ravi Acharya – Artist (image)


**For more information contact :**


**Mr A.Sampath Kumar**
Ph.D Scholar, Department Of Physiotherapy,
Manipal College of Health Professions
Manipal Academy of Higher Education
Ph: +91 8105819191
