# Supplementary material for: Effectiveness of structured exercise program on insulin resistance and quality of life in type 2 diabetes mellitus–A randomized controlled trial
Source: PLoS One. 2024 May 21;19(5):e0302831. doi: 10.1371/journal.pone.0302831 (PMC11108169; doi:10.1371/journal.pone.0302831)
Supplement: S2 File — (PDF) [file pone.0302831.s002.pdf]

Supporting information:

S2: Exercise protocol

Frequency- 3-5days/week

Intensity- 40-85% HRR [Aerobic Exercise], 60-80% 1 RM [Resistance Exercise]

RPE = 12-13/20 or 4-6/10

Type -(A) Aerobic (Brisk Walking) (15-45min)

(B) Resistance exercise program

Major muscles of the upper limb and lower limb

2 sets (1 set = 5–15 repetitions) of

- Biceps curl
- Triceps extension
- Overhead shoulder flexion
- Hip flexion (SLR)
- Quadriceps (Knee extension)
- Hip abduction
- Bridging

Stretching

- Pectorals, Biceps, Hamstrings, Quadriceps, Tendoachilles

Structured exercise program protocol

Parameter	2 weeks	2nd -6th week	6th week-3rd month
Mode	Brisk walking	Brisk walking	Brisk walking
Time	20 minutes	20-35 minutes	35-45 minutes
Intensity	40-55 %	55-65%	65-75%
Frequency	3-5 days	3-5 days	3-5 days

- 10 minutes of warmup
- 10 minutes of cooldown
