# Supplementary material for: Effectiveness of structured exercise program on insulin resistance and quality of life in type 2 diabetes mellitus–A randomized controlled trial
Source: PLoS One. 2024 May 21;19(5):e0302831. doi: 10.1371/journal.pone.0302831 (PMC11108169; doi:10.1371/journal.pone.0302831)
Supplement: S1 Data — (DOCX) [file pone.0302831.s003.docx]

**EFFECTIVENESS OF STRUCTURED EXERCISE PROGRAM ON INSULIN RESISTANCE AND QUALITY OF LIFE IN TYPE 2 DIABETES MELLITUS**

**INTRODUCTION:**

Diabetes mellitus is a chronic metabolic disease characterized by chronic hyperglycemia which is associated with impaired carbohydrate metabolism, lipids and proteins caused by lack of insulin secretion or the tissues decreased sensitivity to insulin metabolic effects. (Motahari-Tabari et al., 2014)

The prevalence of type 2 diabetes is increasing rapidly around the world parallel to the increase in the obesity. In 2011, an estimated 366 million people had diabetes (most of which is type 2) and that number is projected to increase to 552 million by 2030.(Aune, Norat, Leitzmann, Tonstad, & Vatten, 2015)

Diabetes complications are the main causes of morbidity and mortality. It can be prevented by taking medication accurately, following healthy diet and physical activity thus the long term complications will be delayed.(Motahari-Tabari et al., 2014)

Type 2 diabetes is characterized by elevated glucose levels in circulating blood, caused by impairment in glucose tolerance following the development of insulin resistance and relative insulin deficiency. Insulin resistance impair the ability of muscle cells to take up and store glucose and triglycerides, which results in higher levels of glucose and triglycerides circulating in the blood. Impaired glucose control and insulin resistance are reported to be a risk factor for development of cardiovascular disease.(Short et al., 2008)

Insulin resistance (IR) is typically defined as decreased sensitivity and /or responsiveness to insulin-mediated glucose disposal and/or inhibition of hepatic glucose production (HGP).(Gutch, Kumar, Razi, Gupta, & Gupta, 2015) IR plays a major pathophysiological role in type 2 diabetes. It is commonly associated with visceral adiposity, glucose intolerance, hypertension, dyslipidemia, endothelial dysfunction and/or elevated markers of inflammation.(Gutch et al., 2015)

Evaluation of insulin resistance and β-cell function is important for understanding the disease status. The gold standard of evaluation of insulin sensitivity is glucose clamp test.(Okita et al., 2013) The various methods to measure insulin resistance are Hyperinsulinemic euglycemic glucose clamp, oral glucose tolerance test (OGTT) Fasting insulin, Glucose/Insulin ratio, Insulinogenic index (IGI), Homeostatic model assessment , Quantitative insulin sensitivity check index (QUICKI), Minimal model analysis of frequently sampled intravenous glucose tolerance test, Glucose insulin (GI) product, Fasting insulin Resistance Index (FIRI).Most of these methods employed are difficult to apply in clinical practice.(Singh & Saxena, 2010)

Most commonly used measures are Fasting Insulin and Homeostatic Model Assessment –Insulin Resistance (HOMA-IR). Fasting Insulin which is been considered as the most practical approach for the measurement of Insulin Resistance. Homeostatic Model Assessment –Insulin Resistance (HOMA-IR) which is calculated from fasting plasma glucose level and immunoreactive insulin (IRI) is a simple method for evaluation of Insulin resistance.(Singh & Saxena, 2010)

A possible hypothesis is that hyperglycemia, which is an early manifestation in the development of diabetes damages muscle which results in loss if strength and mass. Loss of muscle strength is also an important predictor of physical function and disability in diabetes mellitus. It is associated with excess physical disability in older adults especially in the lower extremity mobility tasks. But the relationship between the diabetes mellitus and loss of muscle strength has not been fully studied.(Kalyani, Metter, Egan, Golden, & Ferrucci, 2015)

Previous studies has reported a decline in thigh muscle cross sectional area (CSA) of 25-40% over the life span of the patient and also seems to be in line with other studies reporting a 30% reduction in leg muscle mass.(Nilwik et al., 2013)

Previous observational studies have described the cross sectional association of fasting and 2 hour post -75 oral glucose tolerance test levels (insulin levels) with loss of muscle mass and strength in person with and without diabetes.(Kalyani et al., 2015)

Insulin resistance would leads to elevated levels of free fatty acids and pro inflammatory cytokines in plasma. Recent advances shows that diabetes mellitus is a pro inflammatory state and the active inflammation would participate in the progression of vascular complication of type 2 diabetes mellitus patients. Currently the inflammation related factors such as high sensitivity C-reactive protein (hs-CRP), Toll-like receptor 2 (TLR2), TLR4, and plasminogen activator inhibitor-1 (PAI-1 ), soluble cell adhesion molecules, interleukin-1b (IL-1b), IL-6, tumor necrosis factor-a and adipocyte derived protein adiponectin have been widely concerned, and their abnormal expression would indicate a pro inflammatory state in a certain degree.(Zhang et al., 2014)

Exercise and physical activity has been considered as a cornerstone for the treatment and prevention of diabetes.(Zou, Cai, Cai, Xiao, & Wang, 2015). There is evidence of a reduced functional capacity in healthy individuals at high risk for development of type 2 diabetes even before the appearance of glucose intolerance. It is well established that patients with a diagnosis of type 2 diabetes have low VO2max values when compared with healthy age-matched controls. Specific pathogenic mechanisms such as hyperglycemia, low capillary density, alterations in oxygen delivery, increased blood viscosity, or presence of vascular and neuropathic complications may also contribute to the decreased VO2max.(Fallis, 2013)

Earlier studies results had showed that participation in regular exercise by people with type 2 diabetes improves blood glucose control, reduces diabetes complications and has favorable effects on cardiovascular events, mortality and quality of life.

Exercise training has long been known as an important non pharmacological tool in the treatment of diabetes.(Lazarevic et al., 2006) The American college of sports medicine presents a strong body of evidence supporting the inclusion of physical activity and exercise in the treatment and management of diabetes. (Fedewa, Gist, Evans, & Dishman, 2014)

Both aerobic and resistance exercise effectively improve insulin sensitivity and lead to better glycemic control in patients with type 2 diabetes. While aerobic exercise has been extensively investigated and shown to be beneficial for glucose lipid metabolism.(Short et al., 2008)

Resistance training has known benefits in older patients with impaired glucose.(Geirsdottir et al., 2012) Previous reviews have established that aerobic and resistance training can be used to improve the regulation of glucose, as well as provide a synergistic effect when combined in a structured exercise program in all ages.(Fedewa et al., 2014)

It is reported that an exercise intervention with 24 week program achieved a beneficial effect on the type 2 diabetes mellitus patients with increased cardiovascular fitness and reduced BMI.(Zou et al., 2015) In an earlier study type and duration of exercise had a greater effect on the results. On the other hand, in most of the studies, the effect of exercise on insulin resistance hasn’t been assessed enough. So, the current study aimed to assess the effects of exercise on insulin resistance and quality of life in type 2 diabetes mellitus.

**REVIEW OF LITERATURE**

**Search strategy:**

A thorough literature search was performed to look into the effectiveness of aerobic, resistance and combined exercise training on insulin resistance in Type 2 Diabetes Mellitus patients. An extensive literature search was done in the following databases: MEDLINE, Pubmed, CINAHL, ProQuest and Scopus and Cochrane database. Key words used were: Type 2 Diabetes Mellitus, Insulin Resistance, Biochemical Markers, Aerobic Exercises, and Resistance Exercises. The key words are used in isolation as well as in combination using Boolean operations ‘AND’ and ‘OR’. Articles were selected from the inception to till March 2016. Full text articles in English language were selected.

**Articles retrieved:**

Randomized controlled trials (RCT): 13

**SUMMARY OF EVIDENCES**

| **Title, author & author** | **Impact of aerobic exercise training on age related changes in insulin sensitivity and muscle oxidative capacity- Kevin R. short et al 2003** | **The effects of 24 weeks of moderate or high intensity exercise on insulin resistance – Gary O Donovan et al 2005** | **A physical activity Programme and its effects on insulin resistance and oxidative defense in obese male patients with type 2 diabetes mellitus- G Lazarevic et al 2006** | **Effect of low intensity exercise therapy on early phase insulin secretion in overweight subjects with impaired glucose tolerance and type 2 DM - Ryoma Michishita 2008** |
| --- | --- | --- | --- | --- |
| **Research objective/ population** | Type 2 diabetes | Type 2 diabetes | Type 2 Dm & obese | Overweight & obese |
| **Study design** | RCT | RCT | RCT | RCT |
| **Sample size** | 90 | 67 | 30 | 30 |
| **Intervention** | 16 week aerobic control or exercise program | 24 weeks – high and moderate intensity exercise | Structured and supervised aerobic exercise program for 6 months | 12 weeks of exercise therapy –submaximal exercise test – bicycle ergometer |
| **Outcome measures** | Insulin sensitivity measured by intravenous glucose tolerance test, bicycle ergometer- vo2 max, treadmill stress test, abdominal fat , fat and fat free mass, body fat, visceral fat, bmi | Venous blood , insulin resistance determined by homeostasis model assessment of insulin resistance- (HOMA-IR) serum triglyceride, o2 consumption- cycling ,respiratory gases – mass spectrometer, vo2 max, polar heart rate monitor, skin fold measurement | Physical activity index, fasting glycaemia, hb1ac, median blood glucose, insulin resistance (HOMA) triglycerides, cholesterol, the Ashwell shape chart health risk, SCORE risk, BMI, waist and hip circumference, blood pressure | Blood samples, anthropometric measurements , sub maximal exercise testing |
| **Main findings** | Exercise training showed improvement in insulin sensitivity | Ex. Training showed significant reduction in insulin resistance & moderate –intensity is as effective as high-intensity exercises | Regular aerobic ex has beneficial effects on glycemic control, insulin resistance , cardiovascular risk, oxidative stress parameters in overweight and obese type 2 diabetes | Improvement seen on impaired glucose tolerance and diabetes mellitus patients after the exercise therapy .insulin sensitivity also improved. |
| **Comments** | 4 months of moderate intensity aerobic exercise improved insulin sensitivity in younger people but not middle aged and older groups. | From this present study suggests that it is also possible to improve glucose metabolism by increasing 3 times/week & one should expend 400 kcal /session at 60% or 80% of maximum capacity. | Regular and structured Programme of moderate physical activity is effective. Group activities under direct supervision are appropriate and beneficial and support the implementation of physical activity into the lifestyle of type 2 DM patients. | Smaller number of subjects , middle aged females and this study helped to demonstrate the low intensity exercise therapy without use of hypoglycemic agents, may possibly improve the β-cell function in subjects with IGT & DM |

| **Title, author & author** | **Aerobic Vs resistance exercise training in modulation of insulin resistance, adipocytokines, and inflammatory cytokine levels in obese type 2 diabetic patients – Sheshab M, Abd El-Kader 2011** | **Effect of 12 week resistance exercise program on body composition, muscle strength , physical function, and glucose metabolism in healthy, insulin resistant, and diabetic elderely Icelanders.-O.G Geirsdottir-2012** | **Changes in insulin resistance and HbA1c are related to exercise-mediated changes in body composition in older adults with type 2 diabetes- Yorgi Mavros et .al 2013** | **Effect of supervised progressive resistance exercise training protocol on insulin sensitivity, glycemia, lipids and body composition in Asian Indians with type 2 dm- Anoop misra et.al 2008** |
| --- | --- | --- | --- | --- |
| **Research objective/ population** | Obese type 2 diabetic patients | Prediabetes and type 2 DM | Older adults with type 2 diabetes | Type 2 diabetes |
| **Study design** | RCT | RCT | RCT | RCT |
| **Sample size** | 40 | 237 | 103 | 30 |
| **Intervention** | Aerobic Vs resistance exercise training- 3 times/week for 3 months | 12 week resistance exercise program -3 times /wk : 3 sets, 6-8 reps. | 12 months of high intensity progressive resistance training- Graded resistance exercise and type 2 DM in older adults (GREAT2DO) | 12 weeks Supervised progressive resistance exercise training protocol |
| **Outcome measures** | Insulin resistance, adipocytokines, tumor necrosis factor, assessment insulin resistance (HOMA), hb1ac. | Body composition, muscle strength, physical function , glucose metabolism , insulin resistant- 6MWT, timed up and go test, isokinetic dynamometer, hydraulic hand held dynamometer | Homeostasis model assessment 2 of insulin resistance (HOMA2-IR) Hb1ac, body composition | Insulin sensitivity , glycaemia, lipids and body composition- short insulin tolerance test, FBG, serum lipids, hsCRP, BMI, dual energy x-ray absorptiometry and CT scans |
| **Main findings** | Aerobic exercise is more appropriate for obese type 2 diabetic patients for modulating insulin resistance, adipocytokines and inflammatory cytokine levels than in resisted ex training. | 12 weeks resistance ex improved the muscle strength and muscle function in prediabetes and type 2 DM | Improvement seen in older adults with type 2 dm achieved only through high intensity PRT | Moderate intensity PRT for 3 months resulted in significant improvement in insulin sensitivity glycaemia, lipids, truncal and subcutaneous adipose tissue |
| **Comments** | Resistance exercise modalities that increases muscle mass may improve glycemic control and insulin resistance. Combined aerobic and resistance exercise improve endothelial vasodilator function and may therefore increase blood flow and glucose uptake in active muscle beds. | In the current study data ,T2DM participants do not experience favorable changes in fasting glucose or Hb1A1c | Future investigations can be done in heterogeneity in body composition adaptations to anabolic exercise in older adults with type 2 DM | Larger sample size , longer duration of study, intense PRT protocol involving more muscle groups should be focused . |

| **Title, author & author** | **The effect of 8 weeks aerobic exercise on insulin resistance in type2 diabetes: a randomized clinical trial - Narges Motahari-tabari et.al 2015** |  |
| --- | --- | --- |
| **Research objective/ population** | Type 2 diabetes |  |
| **Study design** | RCT |  |
| **Sample size** | 53 |  |
| **Intervention** | 8 weeks aerobic exercise- 3 times / week |  |
| **Outcome measures** | Waist and hip circumference, BMI, plasma insulin, HOMA-IR. |  |
| **Main findings** | Aerobic exercises showed improvement on type 2 Dm. these exercise protocol has been effective in lowering plasma glucose, insulin levels and insulin resistance. |  |
| **Comments** | The immediate effect of exercise on glucose and insulin levels after training and change in anti-inflammatory factors during the study were not assessed, which are suggested for further studies |  |

The Literature pertaining to this study will be discussed under the following headings:

1. Effect of exercise training on insulin resistance
2. Physical activity and its effects on insulin resistance
3. Effects of exercise in overweight, obese and type 2 diabetes mellitus
4. Aerobic vs resistance exercise on insulin resistance
5. Diabetes mellitus and quadriceps muscle
6. Diabetes and inflammatory markers
7. **Effect of exercise training on insulin resistance:** Kevin R. Short et.al conducted a study of 16 week aerobic exercise program in healthy men and women aged 21-87 years who exercised <30 min twice per week during the last 9 months were recruited and they concluded that 4 months of moderate intensity aerobic exercises performed by previously sedentary men and women improved insulin sensitivity in young people but not in middle age and older groups.

A study was conducted to investigate the effect of exercise intensity on insulin resistance by comparing moderate and high intensity interventions of equal energy cost. Maximum oxygen consumption (vo2 max) insulin, glucose and triglycerides were measured in 64 sedentary men. They suggested that exercise training is accompanied by a significant reduction in insulin resistance and also suggested that moderate intensity exercise is as effective as high intensity exercises when 400 kcal are expended per session.

A study was done to check the effect of 12 week resistance exercise program on health, insulin resistant and diabetic elderly Icelander patients. They have investigated muscle mass and physical function before and after a resistance exercise program. They concluded that 12 week resistance exercise program improved muscle strength and muscle function in healthy, prediabetes and type 2 diabetes mellitus elderly people.er

Yorgi Mavros et al conducted a study to investigate changes in body composition after 12 months of high – intensity progressive resistance training in relation to changes in insulin resistance or glucose homeostasis in type 2 diabetes. They have given exercises 3 days per week for 12 months. They concluded that exercise showed improvement in body composition which is achieved through high intensity progressive resistance training which also improved insulin resistance and glucose homeostasis in older adults in type 2 diabetes.

Anoop misra et al conducted a study to evaluate the effect of supervised progressive resistance –exercise training protocol on insulin sensitivity, glycaemia, lipids and body composition in Asian Indians with type 2 diabetes. They underwent 12 weeks of progressive resistance training of six muscle groups (2 sets, 10 rep each). They concluded that moderate intensity progressive resistance training for 3 months resulted in significant improvement in insulin sensitivity, glycaemia, lipids in type 2 diabetes.

1. **Physical activity and its effects on insulin resistance:** A study was done to investigate the effects of regular aerobic exercise on glycemic control, insulin resistance, cardiovascular risk and oxidative stress in type 2 diabetic patients. 30 type 2 diabetes patients were assessed for physical activity index, fasting glycaemia, glycated hemoglobin, median blood glucose, insulin resistance triglycerides cholesterol, BMI were compared with 30 healthy control subjects. They concluded that regular aerobic exercise has beneficial effects on glycemic control, insulin resistance, and cardiovascular risk in type 2 diabetes.
2. **Effects of exercise in overweight, obese and type 2 diabetes mellitus:** A study was conducted to evaluate effects of exercise therapy on early phase insulin secretion in overweight subjects with impaired glucose tolerance (IGT) and type 2 diabetes mellitus. All patients performed exercise therapy at lactate threshold intensity for 12 weeks. The results obtained suggested that the beta cell function in subjects with IGT and DM improved after exercise therapy and insulin resistance also improved.
3. **Aerobic vs resistance exercise on insulin resistance:** A study was conducted to compare the impact of aerobic vs resistance training on insulin resistance, adipocytokines and inflammatory cytokine in obese type 2 diabetic patients. 4o obese type 2 diabetic patients were recruited with age ranging from 34-56 years. They concluded that in obese type 2 diabetic patients aerobic exercise is more appropriate for modulating insulin resistance, adipocytokines and inflammatory cytokine levels than is resisted exercise training.
4. **Diabetes mellitus and muscle mass:**  A study was conducted to examine the independent association between diabetes mellitus and quadriceps strength, quadriceps power, and gait speed in national population of older adults. Two thousand five hundred seventy three adults aged 50 and older in the national health and nutrition examination survey 1999-2002 who had assessed the quadriceps strength in USA. They concluded that older U.S adults with diabetes mellitus have lower quadriceps strength and power that is related to the presence of comorbidities and walk slower than those without diabetes mellitus.

A study was conducted in patients with diabetes who have accelerated muscle loss compared with their counterparts. They examined 984 participants aged 25-96 years in the Baltimore longitudinal study of aging (2003-2011) with HbA1c, knee extensor strength (isokinetic dynamometer) and lean body mass (DEXA) measured at baseline. Participants had repeated measurements upto 7.5 years later. They concluded that hyperglycemia is associated with persistently lower muscle strength with aging.

1. **Diabetes and inflammatory markers:** A study was aimed to observe the expression of inflammation-related factors in elderly T2DM patients with or without macrovascular disease (MVD). A total of 64 T2DM patients participated in this study, including 31 patients with MVD (group A) and 33 patients without MVD (group B); and 30 healthy volunteers were recruited as normal control (group C). The levels of serum irisin, retinol binding protein 4 (RBP4) and adiponectin expression were all detected and compared between groups. They concluded that inflammatory disorder is significantly in T2DM patients with MVD, and serum irisin and R BP4 would be reasonable new markers of MVD.

**Research gap:**

- Very few RCT`s with smaller sample size were available in the literature globally.
- Even though prevalence of T2DM increasing rapidly in Indian population there is a dearth of literature on exercise training on insulin resistance in T2DM.

**Need for the study:**

- Even though till date T2DM is highly prevalent & develops IR, there are only few studies available with the effectiveness of exercise on IR.
- IR leads to an exercise intolerance in T2DM, which reduces function & altered quality of life which one again had influence on glycemic control.
- Therefore there is a need to study the exercise intolerance in T2DM.

**AIM OF THE STUDY**

- To find out the effectiveness of structured exercise program on insulin resistance and quality of life in type 2 diabetes mellitus.

**OBJECTIVE OF THE STUDY**

- - To find out the effectiveness of structured exercise program on insulin resistance using fasting insulin level in T2DM.
  - To find out the effectiveness of structured exercise program on quality of life by WHOQOL- BREF.
  - To find out the effectiveness of structured exercise program on functional capacity by six minute walk test.
  - To find out the effectiveness of structured exercise program on glycaemic control by HbA1c.

**CLINICAL SIGNIFICANCE**

- If this study is proved to be effective then structured exercise program can be recommended to reduce insulin resistance in T2DM.

**METHODOLOGY**

**Study design**:

Randomized Controlled Trial

**Study setting**: Department of Medicine, Department of Physiotherapy, Diabetic Clinic, Kasturba Hospital, Manipal, Karnataka, India.

**Participants:**

Type 2 Diabetes Mellitus

**Investigator:**

Physiotherapist pursuing his structured Ph.D. program.

- **Sample size:** Sample size calculated based on the ***Fasting insulin level*** outcome measure

**N= 2[z_1-α/2_ + z_1-β_] ^2^** $\boldsymbol{\times}$**σ^2^**

**(d) ^2^**

***z_1-α/2_ = Level of significance z_1-α/2_ = 1.96***

***z_1-β_ = Power z_1-β =_ 0.84***

***σ = Standard deviation σ=8.26***

***d = Clinical significance difference d = 2***

With a drop out of 20%, the final sample size will be **120** in each group

**Sampling method:** Purposive Sampling

**Randomization:** Block randomization

**Inclusion criteria:**

- Participants with controlled type 2 diabetes mellitus who are on medication.
- Participants with diabetes mellitus whose blood glucose level is above 250 mg/dl will be screened for ketone bodies.
- Aged between 30-65 years of both male & female

**Exclusion criteria:**

- - Participants with type 1 diabetes mellitus
  - Any significant respiratory disease, coronary artery disease, neurological disorders, musculoskeletal problems that would interfere with the exercise training,
  - Pregnancy
  - Thyroid disorder
  - Participants not willing to participate in exercise protocol

**Materials:**

- Bioelectrical Impedence Analysis
- Radio-telemetric heart rate monitor
- Inch tape (non-stretchable)
- Skin fold caliper
- Treadmill
- Weighing scale
- Measuring tape

**Protocol:**

**General guidelines for exercise in Type 2 Diabetes Mellitus:**

Before undergoing any exercise regime a pre-exercise evaluation is required to rule out diabetes related health complications. For individuals desiring to participate in low-intensity physical activity (PA) such as brisk walking, conducting exercise stress testing before walking is unnecessary and moreover no evidence suggests that it is routinely necessary as a CVD diagnostic tool, and requiring it may create barriers to participation among patient population**.**

**AEROBIC & RESISTANCE EXERCISE TRAINING**

| **VARIABLE** | **T2DM** |
| --- | --- |
| Frequency | 3 days / week aerobic exercise  2 days / week of resistance exercise |
| Intensity | 40-85% HRR [Aerobic Exercise] , 60-80% 1 RM [Resistance Exercise]  RPE = 11-16 |
| Time | Aerobic: 30-60 mins per session  Resistance :8-12 reps/ exercise, 1-3 sets/exercise |
| Type of exercise | Aerobic : Brisk Walking , running , cycling  Resistance : All major muscle groups  Upper body: 4-5 exercises  Lower body/core: 4-5 exercises |

**Source: *Acsm Guidelines for Exercise Testing and Prescription 9^th^ edition***

**Consort of Phase – I**

- Permission will be sought from Institutional Research Committee and Institutional Ethical Committee and trial will be registered under CTRI. All participants will be screened for insulin resistance Informed consent will be obtained from the participants who are willing to take part in the study
- Participants will be clinically and biochemically evaluated for following outcomes

**Primary outcome**: Fasting Insulin Level, HOMA-IR, WHOQOL- BREF, Six minute walk test

**Secondary outcome:** Body composition analysis, Fasting blood sugar, GPAQ, HbA1c.

The participants will be randomized into study group and control group (Block Randomization)

Control Group

Study group

No structured exercise program will be given to the control group participants

Study group participants will be given a set of exercise programs along with the standard care.

Standard hospital care will be advised to the participants

Adherence to exercise will assessed by telephonic call, log book and check list will be given to all the participants

Participants will be asked to continue routine work

After 6 weeks progression of exercise program will be prescribed by the principal investigator

Reassessment of all outcome measures will be done at 3 month

Reassessment of all outcome measures will be done at 3 month

Data will be analyzed

**Primary outcome measures:**

- Fasting insulin level [ mIU/L]
- HOMA-IR
- Six minute walk test
- Quality of life by WHOQOL-BREF

**Secondary outcome measures:**

- Body composition analysis
- Fasting blood sugar [mg/dL]
- HbA1c
- Physical activity using GPAQ questionnaire.

**Data analyses:**

- Data will be analysed using SPSS version 15
- Paired t test will be used to analyse within the groups & independent t test is used between the groups
- Descriptive statistics will be used to analyse the age, GPAQ & body composition
- Level of significance will be set at p<0.05

**Research plan schedule:**

| **Study duration** | **1^st^ year** | | | | **2^nd^ year** | | | | **3^rd^ year** | | | | |
| --- | --- | --- | --- | --- | --- | --- | --- | --- | --- | --- | --- | --- | --- |
| **Calendar years** | **2016-17** | | | | **2017-18** | | | | **2018-19** | | | | |
| **Quarterly** | **1** | **2** | **3** | **4** | **5** | **6** | **7** | **8** | **9** | **10** | **11** | **12** |  |
| **Literature review** |  |  |  |  |  |  |  |  |  |  |  |  |  |
| **1. IEC clearance & University registration** |  |  |  |  |  |  |  |  |  |  |  |  |  |
| **2. Phase-I, Randomized controlled trial** |  |  |  |  |  |  |  |  |  |  |  |  |  |
| **5. Data analysis phase-I** |  |  |  |  |  |  |  |  |  |  |  |  |  |
| **6. Reporting and submission** |  |  |  |  |  |  |  |  |  |  |  |  |  |

**References:**

Aune, D., Norat, T., Leitzmann, M., Tonstad, S., & Vatten, L. J. (2015). Physical activity and the risk of type 2 diabetes: a systematic review and dose–response meta-analysis. *European Journal of Epidemiology*, *30*(7), 529–542.

Fallis, A. . (2013). No Title No Title. *Journal of Chemical Information and Modeling*, *53*(9), 1689–1699.

Fedewa, M. V, Gist, N. H., Evans, E. M., & Dishman, R. K. (2014). Exercise and Insulin Resistance in Youth: A Meta-Analysis. *Pediatrics*, *133*(1), E163–E174.

Geirsdottir, O. G., Arnarson, a., Briem, K., Ramel, a., Jonsson, P. V., & Thorsdottir, I. (2012). Effect of 12-week resistance exercise program on body composition, muscle strength, physical function, and glucose metabolism in healthy, insulin-resistant, and diabetic elderly icelanders. *Journals of Gerontology - Series A Biological Sciences and Medical Sciences*, *67*(11), 1259–1265.

Gutch, M., Kumar, S., Razi, S. M., Gupta, K. K., & Gupta, A. (2015). Assessment of insulin sensitivity/resistance. *Indian Journal of Endocrinology and Metabolism*, *19*(1), 160–4.

Kalyani, R. R., Metter, E. J., Egan, J., Golden, S. H., & Ferrucci, L. (2015). Hyperglycemia predicts persistently lower muscle strength with aging. *Diabetes Care*, *38*(1), 82–90.

Lazarevic, G., Antic, S., Cvetkovic, T., Vlahovic, P., Tasic, I., & Stefanovic, V. (2006). A physical activity programme and its effects on insulin resistance and oxidative defense in obese male patients with type 2 diabetes mellitus. *Diabetes & Metabolism*, *32*(6), 583–90.

Motahari-Tabari, N., Ahmad Shirvani, M., Shirzad-e-Ahoodashty, M., Yousefi-Abdolmaleki, E., & Teimourzadeh, M. (2014). The Effect of 8 Weeks Aerobic Exercise on Insulin Resistance in Type 2 Diabetes: A Randomized Clinical Trial. *Global Journal of Health Science*, *7*(1), 115–121.

Nilwik, R., Snijders, T., Leenders, M., Groen, B. B. L., van Kranenburg, J., Verdijk, L. B., & Van Loon, L. J. C. (2013). The decline in skeletal muscle mass with aging is mainly attributed to a reduction in type II muscle fiber size. *Experimental Gerontology*, *48*(5), 492–498.

Okita, K., Iwahashi, H., Kozawa, J., Okauchi, Y., Funahashi, T., Imagawa, A., & Shimomura, I. (2013). Homeostasis model assessment of insulin resistance for evaluating insulin sensitivity in patients with type 2 diabetes on insulin therapy. *Endocrine Journal*, *60*(3), 283–90.

Short, K. R., Vittone, J. L., Bigelow, M. L., Proctor, D. N., Rizza, R. A., Coenen-Schimke, J. M., … Abd El-Kader, S. M. (2008). Effect of supervised progressive resistance-exercise training protocol on insulin sensitivity, glycemia, lipids, and body composition in Asian Indians with type 2 diabetes. *Diabetes*, *67*(7), 179–183.

Singh, B., & Saxena, A. (2010). Surrogate markers of insulin resistance: A review. *World Journal of Diabetes*, *1*(2), 36–47.

Zhang, M., Chen, P., Chen, S., Sun, Q., Zeng, Q. C., Chen, J. Y., … Wang, J. K. (2014). The association of new inflammatory markers with type 2 diabetes mellitus and macrovascular complications: A preliminary study. *European Review for Medical and Pharmacological Sciences*, *18*(11), 1567–1572.

Zou, Z., Cai, W., Cai, M., Xiao, M., & Wang, Z. (2015). Influence of the intervention of exercise on obese type II diabetes mellitus: A meta-analysis. *Primary Care Diabetes*.
